# Vaginal dysbiosis increases risk of preterm fetal membrane rupture, neonatal sepsis and is exacerbated by erythromycin

**DOI:** 10.1186/s12916-017-0999-x

**Published:** 2018-01-24

**Authors:** Richard G. Brown, Julian R. Marchesi, Yun S. Lee, Ann Smith, Benjamin Lehne, Lindsay M. Kindinger, Vasso Terzidou, Elaine Holmes, Jeremy K. Nicholson, Phillip R. Bennett, David A. MacIntyre

**Affiliations:** 1 Imperial College Parturition Research Group, Division of the Institute of Reproductive and Developmental Biology, Imperial College London, London, W12 0NN UK; 2 Centre for Digestive and Gut Health, Imperial College London, London, W2 1NY UK; 3 School of Biosciences, Cardiff University, Cardiff, CF103AX UK; 4 Department of Epidemiology & Biostatistics, Medicine, Imperial College London, London, W2 1PG UK; 5 Chelsea & Westminster Hospital, Imperial College Healthcare NHS Trust, London, SW10 9NH UK; 6 Division of Computational Systems Medicine, Department of Surgery and Cancer, Imperial College London, London, SW7 2AZ UK; 7 Queen Charlotte’s Hospital, Imperial College Healthcare NHS Trust, London, W12 0HS UK

**Keywords:** Vaginal microbiota, Preterm birth, Preterm prelabour rupture of membranes, Antibiotics, Erythromycin, Neonatal sepsis, Pregnancy

## Abstract

**Background:**

Preterm prelabour rupture of the fetal membranes (PPROM) precedes 30% of preterm births and is a risk factor for early onset neonatal sepsis. As PPROM is strongly associated with ascending vaginal infection, prophylactic antibiotics are widely used. The evolution of vaginal microbiota compositions associated with PPROM and the impact of antibiotics on bacterial compositions are unknown.

**Methods:**

We prospectively assessed vaginal microbiota prior to and following PPROM using MiSeq-based sequencing of 16S rRNA gene amplicons and examined the impact of erythromycin prophylaxis on bacterial load and community structures.

**Results:**

In contrast to pregnancies delivering at term, vaginal dysbiosis characterised by *Lactobacillus* spp. depletion was present prior to the rupture of fetal membranes in approximately a third of cases (0% vs. 27%, *P* = 0.026) and persisted following membrane rupture (31%, *P* = 0.005). Vaginal dysbiosis was exacerbated by erythromycin treatment (47%, *P* = 0.00009) particularly in women initially colonised by *Lactobacillus* spp. *Lactobacillus* depletion and increased relative abundance of *Sneathia* spp. were associated with subsequent funisitis and early onset neonatal sepsis.

**Conclusions:**

Our data show that vaginal microbiota composition is a risk factor for subsequent PPROM and is associated with adverse short-term maternal and neonatal outcomes. This highlights vaginal microbiota as a potentially modifiable antenatal risk factor for PPROM and suggests that routine use of erythromycin for PPROM be re-examined.

**Electronic supplementary material:**

The online version of this article 10.1186/s12916-017-0999-x) contains supplementary material, which is available to authorized users.

## Background

Preterm birth and its associated complications are the leading cause of death for children under the age of five worldwide [1]. Survivors often suffer significant motor and sensory deficits, learning disabilities and respiratory disorders [2]. Rupture of the fetal membranes prior to 37 weeks of gestation and before the onset of labour, termed preterm prelabour rupture of the membranes (PPROM), occurs prior to 30% of all spontaneous preterm births [3]. Both pathogenesis of membrane rupture and subsequent maternal and neonatal morbidities are strongly associated with infection [4, 5]. It is hypothesised that colonisation of the vagina with pathogenic bacteria activate the local and upper (cervical and fetal membrane) innate immune system [3, 6], driving an inflammatory cascade [7–10] that leads to remodelling and disruption of membrane architecture and premature rupture [11]. In 80% of cases, delivery occurs within 9 days of rupture [12], during which time the uterine cavity, placenta and fetus are exposed to ascending infection and increased risk of chorioamnionitis and funisitis, which are associated with poor maternal and neonatal outcomes [13–19].

While the vaginal microbiota composition of non-pregnant women is temporally dynamic [20], healthy pregnancy is characterised by a shift towards stable, low-richness and low-diversity community structures dominated by *Lactobacillus* spp. [21–23] that inhibit growth of pathogenic bacteria [24]. However, recent studies have found that the dominance of vaginal bacterial communities by *L. iners* is a risk factor for preterm birth [25, 26]. The absence of *Lactobacillus* spp. and polymicrobial colonisation of the vagina have long been recognised as a risk factor for PPROM [27], preterm birth [28–31] and histological chorioamnionitis [32–34]. Despite a well-described infectious aetiology and high prevalence of chorioamnionitis in PPROM patients, the few studies to have examined vaginal bacterial composition in women with PPROM are limited to small sample sizes collected only after membrane rupture [35–37].

The clinical management of PPROM is challenging, involving an assessment of the balance between prolongation of pregnancy to enable fetal maturation, and risk of infection and subsequent poor neonatal outcomes. As a result, management during this latency period is controversial and varies widely [38]. In many countries, PPROM is managed conservatively with patients receiving steroids to promote fetal lung maturation and prophylactic administration of oral erythromycin at a dose of 250 mg for 10 days [39, 40]. In the presence of clinical evidence of chorioamnionitis, patients are treated with intravenous antibiotics and delivery is expedited [40]. The widespread use of erythromycin for PPROM is based upon the short-term neonatal benefits reported in the ORACLE I trial, which compared erythromycin to co-amoxiclav (amoxicillin and clavulanate potassium) or placebo [41]. The continued use of erythromycin for PPROM is contentious considering the lack of identifiable long-term neonatal benefits [42, 43], limited coverage of gram negative bacteria [44], *Mycoplasma* spp. or *Ureaplasma* spp. [45], rising resistance [46] and recent association with increased risk of cerebral palsy, epilepsy [42, 47] as well as asthma and obesity [48].

Developing a comprehensive understanding of the vaginal microbiota composition and its response to antibiotic treatment in pregnancies complicated by PPROM is, therefore, of paramount importance for improved diagnostic, preventative and therapeutic strategies. In this study, we examined vaginal microbiota compositions prior to and following PPROM, both before and following erythromycin prophylaxis, and correlated these findings with evidence of funisitis and neonatal sepsis.

## Methods

The study was approved by the National Research Ethics Service Committee London–Stanmore of the National Health Service (REC 14/LO/0328), and all participants provided written informed consent.

### Study design

We performed a prospective cohort study whereby women with and without risk factors for preterm birth were recruited between 8 and 12 weeks from the preterm surveillance and antenatal clinics of Queen Charlotte’s and Chelsea Hospital and Chelsea and Westminster Hospital between January 2013 and August 2014 (*n* = 250). Exclusion criteria included women under 18 years of age, multiple pregnancies, sexual intercourse or vaginal bleeding within 72 hours of sampling, and HIV or hepatitis C positive status. Under direct visualisation, cervico-vaginal fluid was sampled from the posterior fornix using a BBL CultureSwab MaxV Liquid Amies swab (Becton, Dickinson and Company, Oxford, UK) at each of the following timepoints: 8–12, 19–25, 27–30 and 32–36 weeks of pregnancy gestation. The vaginal swabs were placed immediately on ice before being snap frozen and stored at -80 °C within 5 min of collection.

A second cohort were recruited upon presentation with ruptured membranes between October 2013 and June 2015 (*n* = 87). As per participating hospitals guidelines, PPROM was defined as a rupture of the fetal membranes, diagnosed by pooling of amniotic fluid on speculum examination, prior to 37 weeks gestation more than 24 hours prior to spontaneous preterm delivery or clinically indicated delivery or induction of labour. Swabs were taken upon presentation before erythromycin treatment, 48 hours after erythromycin treatment, and 1 and 2 weeks post-diagnosis. Patients referred from other hospital sites for specialist neonatal care with a prior diagnosis of PPROM who had already started erythromycin were sampled upon arrival and 1 week later, if undelivered.

All patients were treated conservatively as per the United Kingdom Royal College of Obstetrics and Gynaecology guidelines [40] and the policy of the admitting hospital, which involved receiving antenatal steroids for fetal lung maturation (if less than 34 weeks gestation) and oral erythromycin 250 mg four times a day for 10 days. Delivery was expedited or induced in the presence of fetal distress, clinical signs of chorioamnionitis or at 34 completed weeks of gestation. All patients received intrapartum antibiotics in the form of intravenous benzylpenicillin and the eventual mode of delivery was at the discretion of the attending clinician.

Histological examination of the placenta and fetal membranes was performed following PPROM as per routine practice in the study. Chorioamnionitis was defined as the presence of polymorphonuclear cells within the amnion or chorion whilst funisitis was defined by the presence of polymorphonuclear cells (of fetal origin) within the Wharton’s jelly of the umbilical cord.

Early onset neonatal sepsis (EONS) is defined as the presence of confirmed or suspected sepsis at ≤3 days after birth for which neonatal antibiotic treatment was prolonged beyond the routine 48 hours of prophylaxis. Confirmed sepsis was established by positive blood cultures whilst suspected sepsis was diagnosed in the presence of clinical suspicion of sepsis (lethargy, apnoea, respiratory distress, hypoperfusion and shock) supported by elevated neonatal C-reactive protein (CRP) (>10 mg/dl) or blood film suggestive of bacteraemia. Detailed maternal and neonatal metadata were collected for all participants from the hospital case notes and the electronic patient databases Cerner Millennium®, Powerchart® and Badger.net.

### DNA extraction and 16S rRNA gene sequencing

DNA extraction from vaginal swabs and confirmation of DNA integrity by polymerase chain reaction (PCR) amplification was performed as previously described [22]. The V1–V2 hypervariable regions of 16*S* rRNA genes were amplified for sequencing using forward and reverse fusion primers. The forward primer consisted of an Illumina i5 adapter (5′-AATGATACGGCGACCACCGAGATCTACAC-3′), an 8-base-pair (bp) bar code, a primer pad (forward, 5′-TATGGTAATT-3′), and the 28 F primer (5′-GAGTTTGATCNTGGCTCAG-3′) [49]. The reverse fusion primer was constructed with an Illumina i7 adapter (5′-CAAGCAGAAGACGGCATACGAGAT-3′), an 8-bp bar code, a primer pad (reverse, 5′-AGTCAGTCAG-3′), and the 388R primer (5′-TGCTGCCTCCCGTAGGAGT-3′). Sequencing was performed at RTL Genomics (Lubbock, TX, USA) using an Illumina MiSeq platform (Illumina Inc).

Resulting sequence data were analysed using the MiSeq standard operating procedure pipeline of the Mothur package [50]. Sequence alignment was performed using the Silva bacterial database (www.arb-silva.de/), and classification was performed using the Ribosomal Database Project (RDP) database reference sequence files and the Wang method [51]. The RDP MultiClassifier script was used for determination of operational taxonomic unit taxonomies (phylum to genus) and species-level taxonomies were determined using USEARCH [52]. To avoid sequencing bias, data were resampled and normalised to the lowest read count (*n* = 6940).

Public access to sequence data and accompanying metadata can be obtained from the Sequence Read Archive of the European Nucleotide Archive (PRJEB21325).

### Quantitative bacteriology

The total number of 16S rRNA gene copies per swab was measured as a representation of the total bacterial load. A bacterial DNA template was used for broad coverage quantitative real-time PCR using the BactQUANT method [53]. For this, a tenfold standard curve (30 to 3,000,000 copies) of *Escherichia coli* 16S DNA (Sigma, D4889) was generated and each standard was combined with 5 μl of sample DNA templates and platinum PCR-supermix UDG containing 50 nM Rox (Life Tech, cat. no. 11730-017), BactQUANT forward primer sequence (5' CCT ACG GGA GGC AGC A), BactQuant reverse primer sequence (5' GGA CTA CCG GGT ATC TAA TC) and BactQUANT probe (Life Tech, cat. no. 4316034, 6000pmol scale) sequence 5' 6FAM-CAG CAG CCG CGG TA-MGBNFQ. Template-free PCR controls and sham digest controls were included in each run. Bacterial load was displayed as copy number per swab corrected for variation of 16S rRNA gene copy proportional to bacterial species abundance in each swab. For this, 16S rRNA gene copy number of bacterial species comprising >95% of sequence reads for each swab was identified using the Michigan rrn database (https://rrndb.umms.med.umich.edu/) and weighted for relative abundance. Bacterial load values were then normalised to the weighted copy number. Where operon copy number was not available at the species level, average copy number at the genera level was used.

### Statistical analysis

Examination of statistical differences between vaginal microbiota was performed at genera and species taxonomic levels using the Statistical Analysis of Metagenomic Profiles software package (STAMP) [54]. Samples were classified into eight vaginal microbiota groups (VMGs) according to Ward’s linkage hierarchical clustering analysis of bacterial species using a clustering density threshold of 0.75 with the 50 most abundant species displayed. Clusters were then sub-grouped based on *Lactobacillus* abundance into *Lactobacillus* dominant (>80%), intermediate abundance (33–78%) and depleted/dysbiotic (<10%).

The significance of differences between richness and diversity measures, bacterial load and relative abundance of species and genera data between patient groups was assessed using one-way ANOVA with Dunn’s multiple comparisons and the Mann–Whitney *t*-test where appropriate.

The linear discriminant analysis with effect size (LEfSe) method [55] was used to identify differentially abundant taxonomic features between patient groups of interest. An α value of 0.05 was used for the factorial Kruskal–Wallis test between classes and a minimum threshold of 2.0 was used for the logarithmic latent discriminatory analysis (LDA) score for discriminative features for all LEfSe plots.

Transition of the VMGs following administration of erythromycin was visualised using a Sankey plot created in the JavaScript Sankey Diagram package from Google Charts (https://developers.google.com/chart/interactive/docs/gallery/sankey). A statistical comparison of data from paired samples obtained before and after erythromycin was administered was performed using a Wilcoxon signed rank test.

To assess the statistical significance of dysbiosis and microbiome groups, we performed linear regression analysis in the R programming environment. Specifically, we used the function lmer() (R package lme4 version 1.1-7, http://CRAN.R-project.org/package=lme4) where paired samples were present and lm() where no paired samples were present. For each analysis, a false discovery rate adjustment (Benjamin and Hochberg) was applied to correct *P* values. In total, four analyses were carried out as follows:

(i) Analysis of differences in microbiome composition between patient groups and dysbiosis/microbiome groups. An indicator variable is created, where the indicator is 1 for samples that could be assigned to the given dysbiosis or microbiome group and the indicator is 0 for all other samples. This indicator variable is regressed against pairs of patient groups adjusted for maternal age, ethnicity, body mass index, smoking status, cervical stitch and progesterone treatment. Where paired samples were available, the model also included the patient ID modelled as a random effect.
(ii) Analysis of differences in microbiome composition between PPROM patients (*n* = 16) before erythromycin was given and 48 hours thereafter. As above, an indicator variable is created and regressed against the time point. As all samples are paired, no additional predictors are included in the model.
(iii) Analysis of differences in microbiome composition between healthy patients and patients with chorioamnionitis or funisitis. As above an indicator variable is created and regressed against the chorioamnionitis/funisitis status adjusted for maternal age, ethnicity, body mass index, smoking status, cervical stitch, progesterone usage and latency.
(iv) Analysis of differences in microbiome composition between PPROM patients with and without EONS. As above, an indicator variable is created and regressed against the EONS status adjusted for maternal age, ethnicity, body mass index, smoking status, cervical stitch and progesterone usage.

## Results

### Baseline clinical characteristics of the study cohort

A total of 250 pregnant women were prospectively recruited while attending a prematurity surveillance clinic after a history of preterm birth or mid-trimester loss and followed up at 8–12, 19–25, 27–30 and 32–36 weeks of gestation. The majority of these patients delivered at term (*n* = 212, 85%). Of those who delivered preterm after <37 weeks of gestation (38/250, 15%), 15 experienced PPROM and were sampled at a mean gestational age of 30^+1^ weeks. Samples collected at 27–30 weeks (mean 29 weeks) from 20 patients who delivered at term following uncomplicated pregnancy were used as gestational age matched controls. An additional 87 women presenting acutely with PPROM at a mean gestational age of 28+1 weeks were recruited; 39 were sampled before erythromycin prophylaxis, 48 after erythromycin only. Of these women, 16 were sampled both before and after erythromycin treatment.

There was no significant difference in maternal age (*P* = 0.93, Mann–Whitney) or ethnicity (*P* = 0.92, Fisher’s exact) between prospectively recruited control or PPROM cases or women presenting with PPROM (Table 1). Women who were recruited after PPROM had membrane rupture at an earlier gestational age than those who were sampled prior to rupture (27^+0^ vs. 34^+4^ weeks, *P* = 0.0011; Mann–Whitney). Gestational age at delivery (30^+4^ vs. 34^+4^ weeks, *P* = 0.0142), 1-minute post-delivery Apgar scores (6 vs. 9, *P* = 0.004) and birth weight (1550 g vs. 2285 g, *P* = 0.0194, Mann–Whitney) were lower in this cohort compared to prospectively sampled women subsequently experiencing PPROM, whereas admissions to the neonatal unit were higher (68% vs. 20%, *P* = 0.00094, Fisher’s exact).

**Table 1:** Clinical and demographic characteristics of the study population

	Uncomplicated term delivery	Before PPROM	*P* value (UTC vs. before PPROM)	After PPROM	*P* value (before PPROM vs. after PPROM)	*P* value all
Total number	20	15		87		
Age (years)	33 (31–34)	34 (30–37)	0.6410	33 (32–34)	0.79	0.9279
Ethnicity *n* (%)						
Caucasian	10 (50%)	6 (40%)		38 (44%)		
Black	5 (25%)	3 (20%)	0.6393	20 (23%)	0.8796	0.9207
Asian	5 (25%)	6 (40%)		29 (33%)		
GA first sample (weeks)	28^+2^ (28^+1^–29^+0^)	32^+0^ (27^+0^–34^+0^)	0.1871	28^+1^ (25^+1^–33^+0^)	0.2743	0.4504
GA at MR (weeks)	n/a	34^+4^ (31^+0^–35^+2^)		27^+0^ (24^+6^–32^+4^)	0.0011	
GA at delivery (weeks)	40^+1^ (39^+2^–41^+1^)	34^+4^ (31^+0^–35^+2^)	6.12 × 10^-7^	30^+4^ (26^+5^–34^+2^)	0.0142	<0.0001
Apgar score						
1 min	9 (8–9)*	9 (8–9)*	0.6432	6 (5–9)**	0.0013	<0.0001
5 min	10 (10–10)*	9 (9–10)*	0.0656	8 (7–9)**	0.0183	<0.0001
BW (g)	3400 (3215–3980)*	2285 (1720–2560)	0.000005	1550 (877.5–2216)	0.0140	<0.0001
Admission to neonatal unit	0 (0%)	3 (20%)	0.069519	59 (68%)	0.00094	<0.0001
Latency						
PPROM to delivery (days)	n/a	1 (0–4)		4 (2–9)	0.0212	

Data presented as median (interquartile range) or number (%)

*BMI* body mass index, *BW* birth weight, *GA* gestational age, *MR* membrane rupture, *n/a* not applicable, *PPROM* Preterm prelabour rupture of the fetal membranes

*P* values: *t*-test/Mann–Whitney U (depending upon distribution), Fisher’s exact for proportional data, ANOVA for multiple comparisons

Missing data * *n* = 5, **n = 15

### Vaginal microbiota composition prior to and following PPROM

A total of 4,542,391 high quality reads were generated in the study, with a mean read count per sample of 27,530 (range 6940 to 163,610). After removing singletons and rare operational taxonomic units (<10 reads per sample), a total of 37, 84 and 332 taxa were identified in vaginal swabs collected from controls, women who subsequently developed PPROM and women sampled at time of PPROM presentation, respectively.

**Fig. 1:**
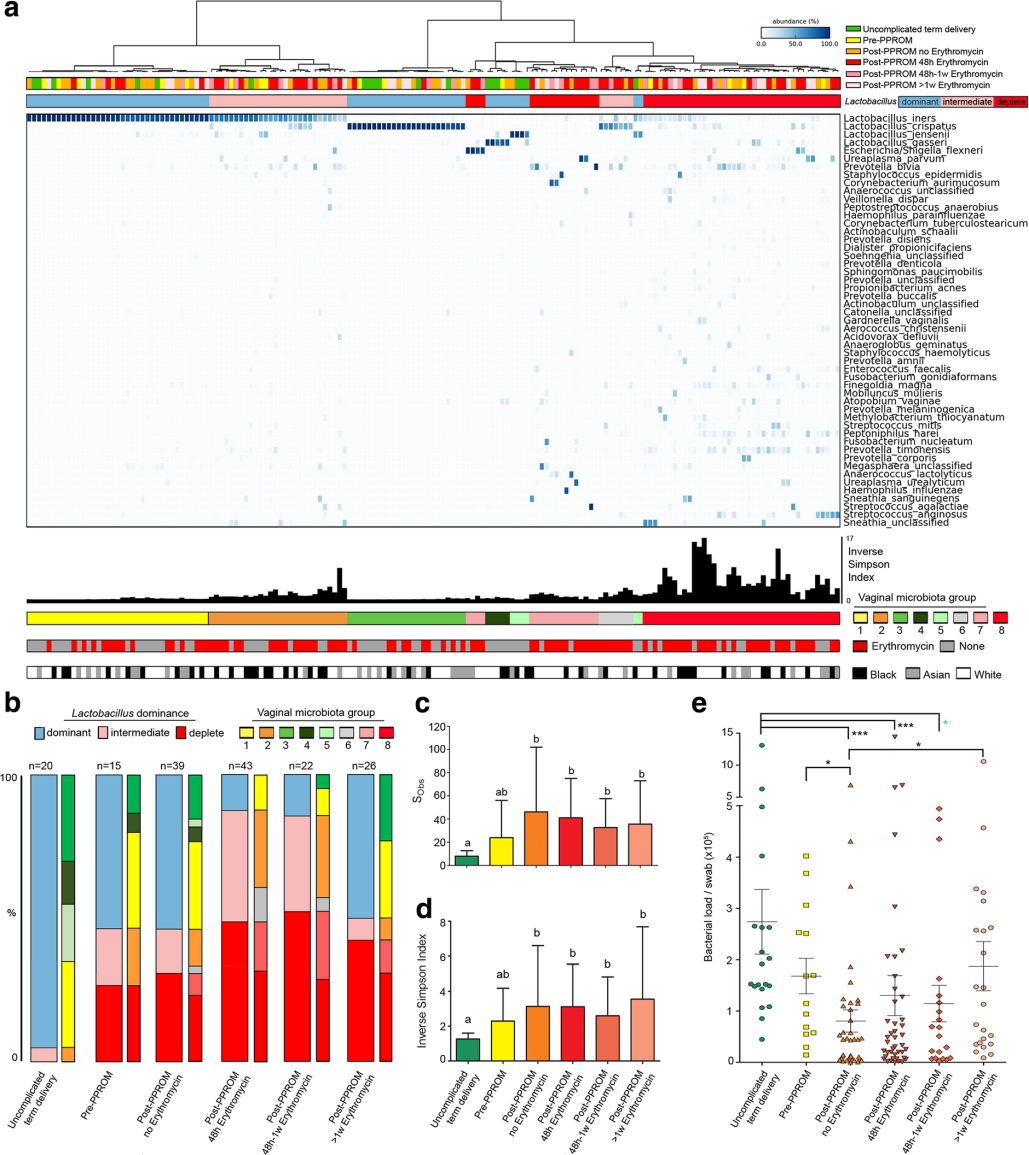
Decreased vaginal *Lactobacillus* spp. abundance occurs prior to PPROM and is further exacerbated by membrane rupture and erythromycin treatment. **a** Ward’s linkage hierarchical clustering analysis of vaginal bacterial species data from cervical vaginal fluid samples (*n* = 165) collected from women with uncomplicated term delivery, sampled at 28 weeks (*n* = 20), pre-PPROM (*n* = 15), following PPROM before erythromycin (*n* = 39), after 48 hours of erythromycin (*n* = 43), 48 hours to 1 week of erythromycin (*n* = 22) and >1 week of erythromycin treatment (*n* = 26). Vaginal bacterial communities were classified based on *Lactobacillus* spp. abundance into dominant, intermediate and depleted, and further into eight vaginal microbiota groups: VMG 1: *L. iners* dominant, VMG 2: *L. iners* high diversity, VMG 3: *L. crispatus* dominant, VMG 4: *L. gasseri* dominant, VMG 5: *L. jensenii* dominant, VMG 6: *L. crispatus* high diversity, VMG 7: *Lactobacillus* spp. depleted and low diversity, VMG 8: *Lactobacillus* spp. depleted and high diversity. **b** Relative *Lactobacillus* spp. abundance is significantly lower in the pre-PPROM and membrane rupture groups compared to gestation age matched and normal pregnancy controls (*P* = 0.011). Erythromycin treatment exacerbates *Lactobacillus* spp. depletion and expansion of dysbiotic vaginal communities (*P* = 0.001). Reduced *Lactobacillus* spp. abundance is accompanied by a reciprocal increase in **c** richness and **d** diversity. **e** Bacterial load is significantly higher pre-membrane rupture in comparison to post-membrane rupture (*P* = 9.37 × 10^-8^) but remains stable thereafter, despite ongoing erythromycin treatment. PPROM preterm prelabour rupture of the fetal membranes

Samples were classified into eight VMGs using hierarchical clustering of relative abundance data from the top 50 bacterial species identified (accounting for >95% of all sequence reads) (Fig. 1). VMGs 1, 3, 4 and 5 were characterised by the dominance of *Lactobacillus* species: *L. iners* (>92% relative abundance, VMG 1), *L. crispatus* (>93%, VMG 3), *L. gasseri* (>80%, VMG 4) and *L. jensenii* (>92%, VMG 5), consistent with previous descriptions of vaginal bacterial communities in non-pregnant [56, 57] and pregnant populations [22, 23]. VMGs 2 and 6 were characterised by reduced *L. iners* (33–68%) and *L. crispatus* (51–78%) dominance, respectively, and significantly elevated diversity and richness (Additional file 1: Figure S1). VMG 7 was characterised by low relative abundance of *Lactobacillus* spp. (<1%, VMG 7) and low diversity. VMG 8 had low *Lactobacillus* spp. levels (<10%) but high diversity and richness (Additional file 1: Figure S1 and Table S1). VMGs were alternatively grouped according to relative abundance of *Lactobacillus* spp. into dominant (VMGs 1, 3, 4 and 5), intermediate (VMGs 2 and 6) and depleted (VMG 7 and 8) categories.

Vaginal microbial communities isolated from control women sampled at 28 weeks of gestation were characterised by low richness and diversity and dominance by *Lactobacillus* spp. (Fig. 1a–d). In contrast, women sampled antenatally who subsequently experienced PPROM had a higher proportion of microbiota profiles characterised by intermediate or low *Lactobacillus* spp. dominance and high diversity (7/15, *P* = 0.011, Fisher’s exact). Significant differences in the proportion of *Lactobacillus* spp. dominant, intermediate and depleted communities remained when analyses were adjusted for potential confounders, including maternal age, ethnicity, body mass index, smoking status and treatment interventions (cervical stitch or progesterone therapy) (Additional file 1: Table S2).

**Fig. 2:**
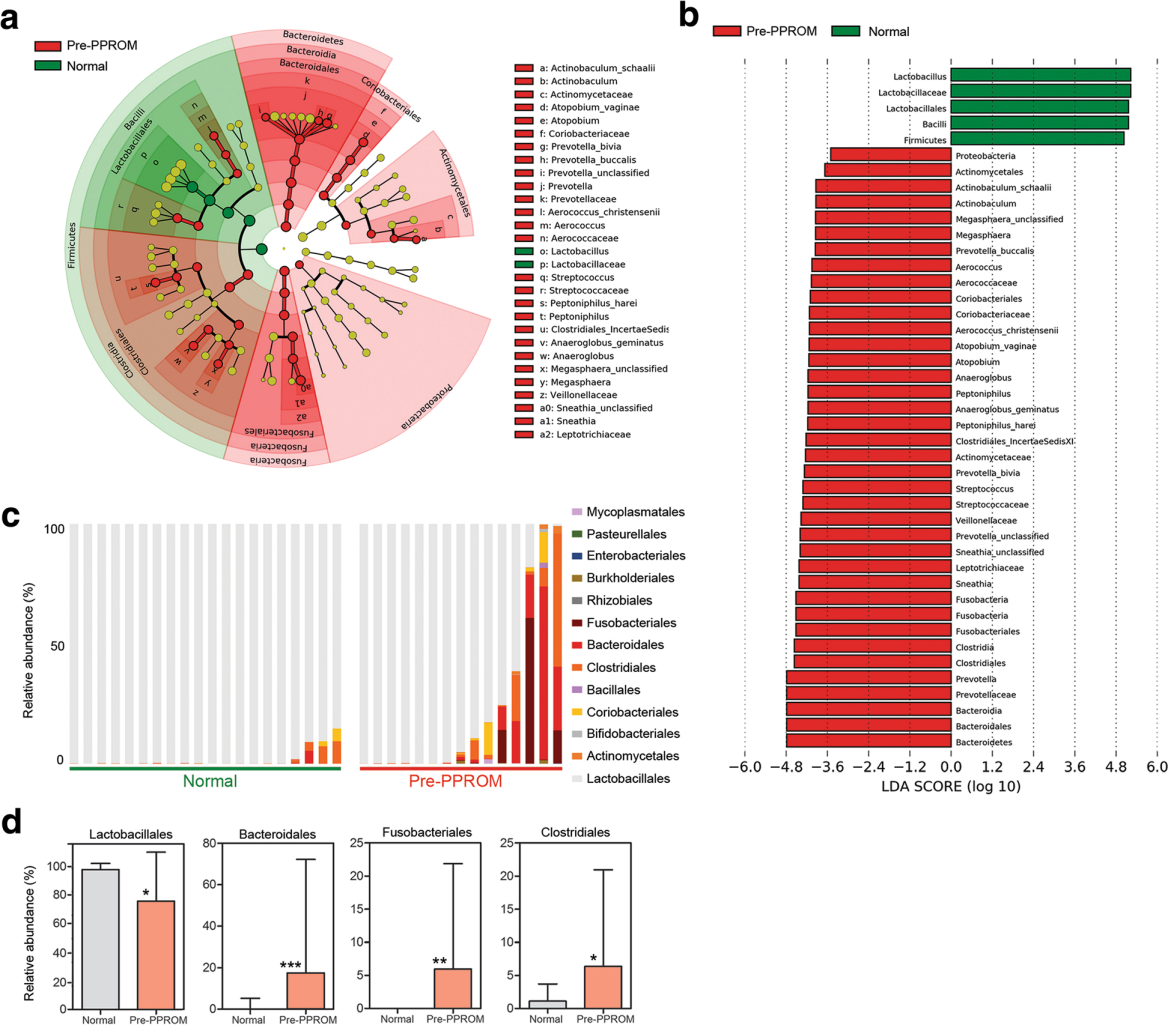
Bacterial taxonomic groups discriminate between normal-term delivery and women destined to experience PPROM. **a** Cladogram describing differentially abundant vaginal microbial clades and nodes observed between women subsequently experiencing normal-term delivery or PPROM as identified using LEfSe analysis. **b** The effect size for each differentially abundant species was estimated using LDA. Vaginal microbiota of patients prior to PPROM was enriched with Bacteroidales, Fusobacteriales and Clostridiales whereas those with a term delivery were comparatively enriched in Lactobacillales. **c** Stacked bar charts of relative abundance for each individual sampled highlight the emergence of a high-diversity microbial profile and reduced dominance of the *Lactobacillus* genus. **d** Comparison of relative abundance across the four differentially expressed bacterial genera showing reduced Lactobacillales (*P* = 0.0172) and increased Fusobacteriales (*P* = 0.0035), Clostridiales (*P* = 0.0356) and Bacteroidales (*P* = 0.009, Mann–Whitney U, two-tailed) in women prior to membrane rupture compared to controls. LDA latent discriminatory analysis, LEfSe linear discriminant analysis with effect size, PPROM preterm prelabour rupture of the fetal membranes

To identify vaginal bacteria associated with PPROM risk, we used LEfSe on 16S rRNA data collected from control patients and women sampled antenatally prior to PPROM. Samples from the latter cohort were enriched in bacteria classes Bacteroidales, Fusobacteriales and Clostridiales, whereas increased Lactobacillales was predictive of normal-term delivery (Fig. 2a–d).

### Impact of membrane rupture on the vaginal microbiome

Membrane rupture prior to erythromycin prophylaxis was associated with a reduction in bacterial load compared to both control (*P =* 9.37 × 10^-8^) and PPROM cases (*P* = 0.0011) sampled prior to membrane rupture (Fig. 1e). Although a similar proportion of *Lactobacillus* spp. depleted, high-diversity communities were observed post-rupture (Fig. 1b), membrane rupture was associated with a significant increase in richness (*P* = 0.000001, Fig. 1c) and alpha diversity (*P* = 0.00194, Fig. 1d). LEfSe analysis identified 20 genera positively associated with membrane rupture (Additional file 1: Figure S2) including *Prevotella*, *Staphylococcus*, *Aerococcus* and *Streptococcus* spp., and a negative association with *Lactobacillus* spp.

### The effect of erythromycin treatment on vaginal microbiota following PPROM

**Fig. 3:**
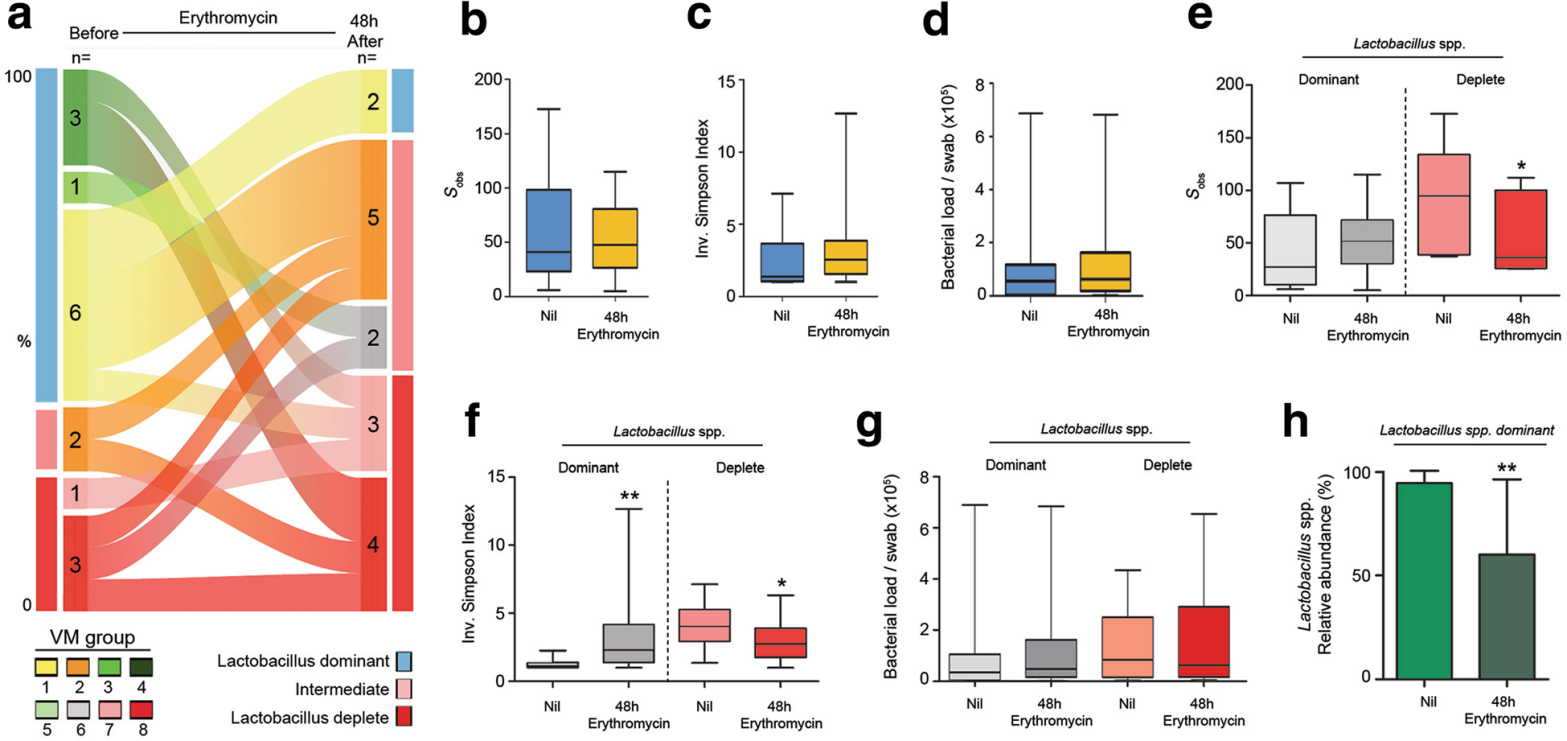
Erythromycin treatment promotes vaginal dysbiosis. **a** Analysis of vaginal microbiota communities from paired samples before and following 48 hours of erythromycin treatment demonstrates transition from *Lactobacillus* spp. dominance (*n* =  = 10, 63%) towards intermediate (*n* = 4) or complete *Lactobacillus* spp. depletion (*n* = 2) whilst communities initially low in *Lactobacillus* spp. remain so. Erythromycin treatment did not reduce **b** richness, **c** diversity or **d** bacterial load. A sub-analysis showed that erythromycin treatment was associated with decreased **e** richness (*P* = 0.03) and **f** diversity (*P* = 0.03) in communities initially depleted in *Lactobacillus* spp., treatment but was associated with a significant increase in diversity (*P =* 0.01) in communities initially dominated by Lactobacillus spp. **g** Bacterial load was similar between subgroups. **h** *Lactobacillus* spp. abundant communities experienced a 40% reduction in *Lactobacillus* spp. post treatment (*P* = 0.004, Wilcoxon signed rank test). Inv. inverse, VM vaginal microbiota

Analysis of cross-sectional samples taken after PPROM following 48 hours of oral erythromycin treatment (average 8 × 250 mg doses) demonstrated a strong shift towards dysbiosis as characterised by a reduction in the proportion of *Lactobacillus* spp. dominated vaginal microbiota and an increase in intermediate communities (Fig. 1b, *P* = 0.001, Fisher’s exact, Additional file 1: Table S2) that persisted for up to 1 week of treatment. Treatment beyond 1 week was associated with a reduction of intermediate communities and an increase in *Lactobacillus* spp. dominance. However, the proportion of dysbiotic vaginal microbiota remained constant. Bacterial richness (Fig. 1c), diversity (Fig. 1d) and load (Fig. 1e) remained unchanged following erythromycin treatment at all time points. Similar results were observed in paired samples taken before and 48 hours after erythromycin treatment (*n* = 16, Fig. 3a–d). A sub-analysis of women with *Lactobacillus* spp. dominance prior to erythromycin exposure (*n* = 10) showed that treatment was associated with a shift towards an intermediate or dysbiotic community structure in 80% of cases (8/10, *P* = 0.009, Fisher’s exact, Fig. 3a, Additional file 1: Table S3), a significant decrease in *Lactobacillus* spp. relative abundance (*P* = 0.0039, Wilcoxon matched pairs, Fig. 3h) and increased diversity (*P* = 0.0098, Wilcoxon matched pairs) (Fig. 3e,f). In contrast, erythromycin treatment was associated with a reduction in both richness (*P* = 0.031) and diversity (*P* = 0.031, Wilcoxon matched pairs) in samples collected from women with *Lactobacillus* spp. depletion prior to treatment despite unchanged bacterial load (Fig. 3g).

### Relationship between vaginal microbiota composition and chorioamnionitis with funisitis

Histopathologic examination of placenta, fetal membranes and umbilical cord was performed following delivery for 53 PPROM cases (53/87, 61%). Evidence of chorioamnionitis with funisitis, chorioamnionitis or normal histology were reported in 34 (64%), 4 (8%), and 15 (28%) cases respectively. Cases of chorioamnionitis and chorioamnionitis with funisitis were combined for further analyses. The average gestational age for membrane rupture in women with chorioamnionitis +/- funisitis was similar to those with normal histology [26^+3^ (25^+3^–27^+2^) vs. 27^+1^ (25^+3^–28^+6^) gestation weeks_,_ *P* = 0.43, Mann–Whitney test]. The average latency between membrane rupture and delivery was not statistically different between groups [6.8 (5–8.7) days for those with chorioamnionitis +/- funisitis vs. 24.7 (6.2–43.2) days for normal histology, *P* = 0.88, Mann–Whitney], gestational age at delivery was significantly lower for those cases complicated by chorioamnionitis +/- funisitis [27^+3^ (26^+3^–28^+2^) vs. 31^+5^ (29^+4^–33^+6^) gestational weeks, *P* = 0.009, Mann–Whitney, Additional file 1: Table S4].

**Fig. 4:**
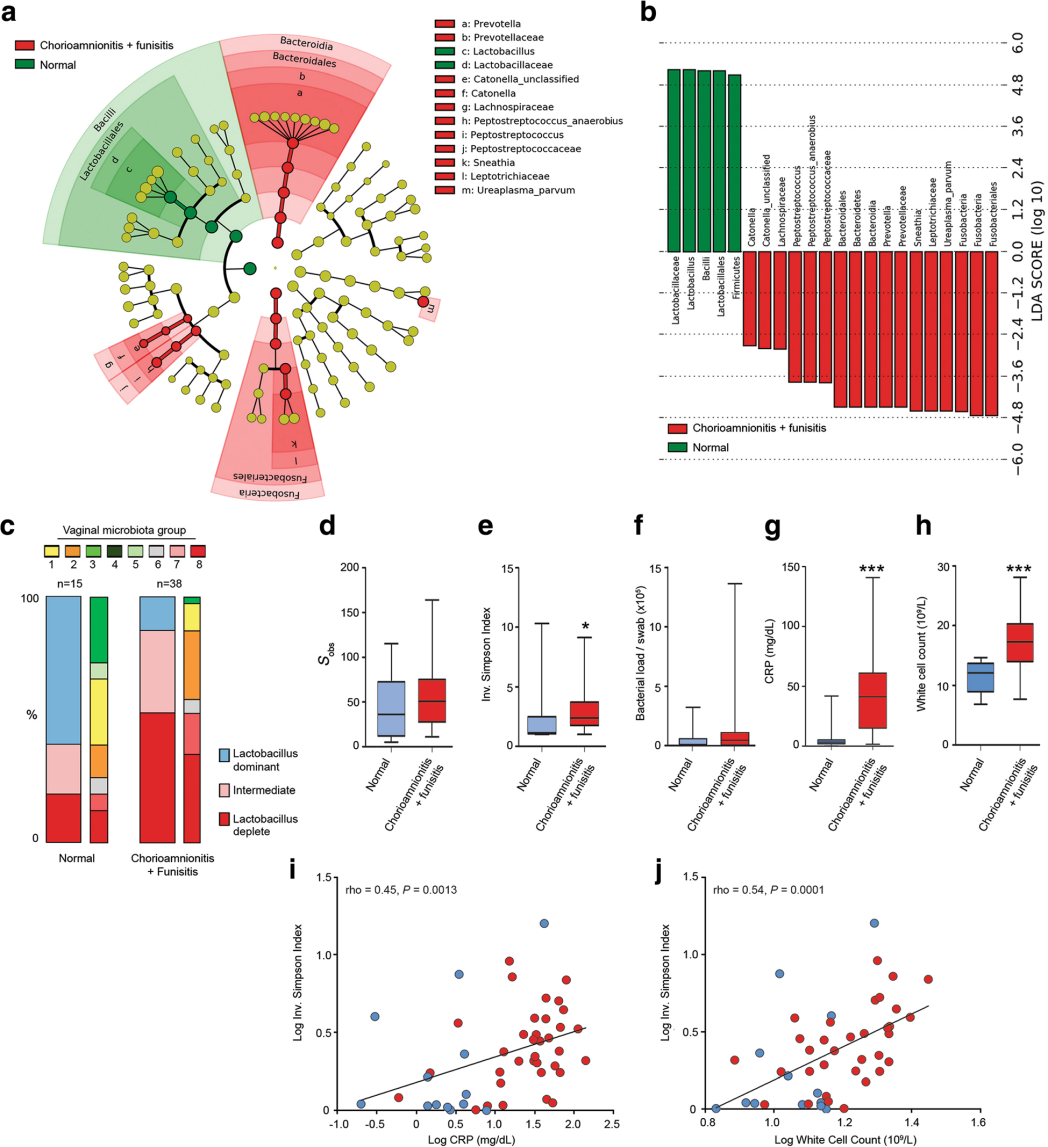
Vaginal dysbiosis is associated with increased risk of chorioamnionitis with funisitis following PPROM. **a** Differentially abundant vaginal taxa detected using LEfSe analysis in samples taken prior to delivery in women with normal histology or chorioamnionitis with funisitis. **b** The vaginal microbiome of patients with normal histology was enriched with *Lactobacillus* spp. whereas those with chorioamnionitis with funisitis were enriched with Fusobacteriales including *Sneathia*, *Bacteroidales*, *Peptostreptococcus* and *Catonella* species. **c** Increased prevalence of *Lactobacillus* depleted/dysbiotic communities with significantly reduced *Lactobacillus* abundance was observed in the chorioamnionitis with funisitis cohort (*P* = 0.0025, Fisher’s exact). **d** No difference was detected in vaginal microbial richness between groups. However, **e** diversity was increased in cases complicated by chorioamnionitis with funisitis. **f** Bacterial load was comparable between histological groups. **g**, **h** Serum markers of maternal infection and inflammation were significantly elevated in cases of chorioamnionitis, +/- funisitis (CRP: *P* = 0.000016, WCC: *P* = 0.0016). **i**, **j** Both WCC (rho = 0.54, *P* = 0.0001) and CRP (rho = 0.45, *P* =0.0013) were positively correlated with vaginal bacterial alpha diversity. CRP C-reactive protein, LDA latent discriminatory analysis, LEfSe linear discriminant analysis with effect size; WCC white cell count

Examination of vaginal microbiota composition in samples obtained just prior to delivery showed that chorioamnionitis with funisitis was associated with enrichment for *Prevotella*, *Sneathia*, *Peptostreptococcus* and *Catonella* spp. and reduced *Lactobacillus* spp. levels (*P* = 0.0025) compared to women with normal histology (Fig. 4a–c, Additional file 1: Table S5). Chorioamnionitis with funisitis was associated with increased vaginal alpha diversity (*P* = 0.0134, Mann–Whitney, Fig. 4e), but not richness (Fig. 4d) or bacterial load (Fig. 4f). Maternal CRP (*P* = 0.000016) and white cell count (WCC) (*P* = 0.0016, Mann–Whitney U) (Fig. 4g,h) were elevated in patients with chorioamnionitis with funisitis and both were significantly correlated with vaginal bacterial alpha diversity (WCC; rho = 0.54, *P* = 0.0001 and CRP; rho = 0.45, *P* = 0.0013) (Fig. 4i,j).

### Vaginal microbiota and early onset neonatal sepsis

**Fig. 5:**
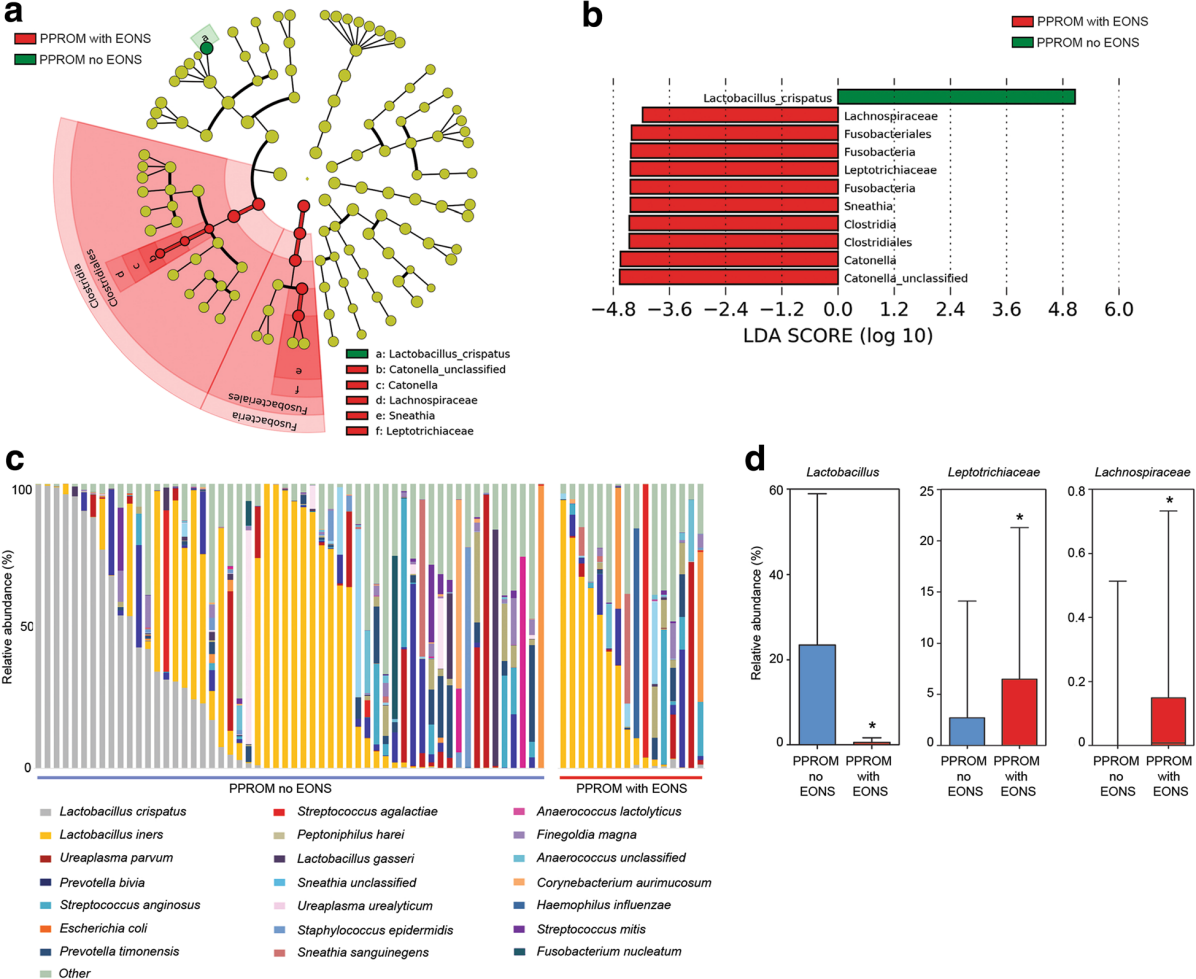
Vaginal dysbiosis is a risk factor for early onset neonatal sepsis (EONS) following PPROM. **a** Cladogram and **b** LDA of differentially abundant species detected in vaginal swab samples collected just prior to delivery in cases who did or did not subsequently develop EONS. *Lactobacillus crispatus* was void from EONS samples, which were enriched with bacteria from genera *Sneathia* and *Catonella*. **c** Distribution of the 21 most abundant bacterial species across the two patient groups. *Lactobacillus crispatus* was present only in mothers whose babies did not develop EONS. **d** Relative abundance of differentially expressed genera: *Lactobacillus* (*P* = 0.001), *Leptotrichiaceae* (*P* = 0.0291) and *Lachnospiraceae* (*P* = 0.0114). EONS early onset neonatal sepsis, LDA latent discriminatory analysis, PPROM preterm prelabour rupture of the fetal membranes

A total of 16 cases of EONS (22%, 16/72) were identified from the cohort once intrauterine deaths (*n* = 2) and cases with insufficient or missing neonatal metadata were removed (*n* = 13). Neonatal CRP (*P* = 1.79 × 10^-8^, Mann–Whitney), duration of neonatal antibiotics (118 hours vs. 44 hours, *P* = 3.12 × 10^-8^, Mann–Whitney), maternal CRP (*P =* 0.0008, Mann–Whitney U) and presence of chorioamnionitis +/- funisitis (*P* = 0.0051, chi-squared) were all significantly higher compared to PPROM cases without EONS. Gestational age at delivery [31^+2^ (30^+1^–32^+2^) vs. 27^+6^ (26^+1^–29^+3^) weeks of gestation, *P* = 0.0046, Mann–Whitney], birth weight [1707 (1519–1896) vs. 1100 (790–1411) g, *P* = 0.0037, Mann–Whitney] and Apgar scores at 1 (*P* = 0.046), 5 (*P* = 0.01) and 10 minutes (*P* = 0.03, all Mann–Whitney) were significantly lower in the EONS group. Neonatal gender, mode of delivery, arterial cord pH and latency between membrane rupture and delivery were comparable (Additional file 1: Table S6). Vaginal swabs collected closest to the time of delivery were enriched for *Catonella* spp*.* and *Sneathia* spp. in cases developing EONS, whilst *Lactobacillus crispatus* was overrepresented in the maternal vaginal microbiota of neonates who did not develop EONS (Fig. 5a–d). Other species previously associated with EONS, including *Streptococcus agalactiae*, *Fusobacterium nucleatum* and *Escherichia coli*, were frequently observed in vaginal samples collected from EONS-complicated pregnancies but not in samples isolated from uncomplicated controls (Fig. 4c). Similar results were obtained when the analysis was repeated only for those mothers who delivered at 28 weeks or sooner (*n* = 27) (Additional file 1: Figure S3).

## Discussion

Infection is strongly associated with PPROM and as a result, empiric antibiotic therapy is routinely used, particularly in high-income countries [58]. In this study, we show that vaginal dysbiosis is present prior to the rupture of fetal membranes in approximately a third of cases and is associated with both chorioamnionitis with funisitis and with EONS. Reported benefits of antibiotic treatment following PPROM are often attributed to the prevention of neonatal infection caused by ascending colonisation of pathogenic bacteria originating from the vagina [59]. We, therefore, hypothesised that prophylactic erythromycin would lead to a reduction of vaginal bacterial load, diversity and richness. However, treatment was associated with a shift towards vaginal dysbiosis, particularly in women initially colonised predominately by *Lactobacillus* species. Our sub-analysis showed that in women with *Lactobacillus* spp. dominance, erythromycin exposure was associated with a shift towards a dysbiotic community structure in most cases. In contrast, erythromycin treatment was associated with a reduction in both richness and diversity in women with a *Lactobacillus* spp. depleted vaginal microbiota. There are, therefore, two groups of women who experience PPROM, for one of which erythromycin therapy is detrimental and for the other potentially beneficial. This has important implications for the continued use of prophylactic erythromycin in the context of PPROM, as is currently recommended by the World Health Organization (WHO) [60] and professional bodies throughout the world including the United Kingdom [40], Canada [61], Germany [62], Australia and New Zealand [63].

*Lactobacillus*-depleted high-diversity vaginal bacterial communities have been identified as risk factors for preterm birth in prospective studies using both culture-dependent [29, 64] and culture-independent [30, 31] methods. The pathophysiology linking vaginal dysbiosis to activation of inflammation and untimely stimulation of prolabour pathways in gestational tissues is well documented [6, 65, 66]. Our results indicate that around one third of patients have vaginal dysbiosis prior to membrane rupture, providing further evidence for ascending vaginal infection in the pathophysiology of PPROM and preterm birth. Haematogenous infection of the gestational tissues leading to rupture may be responsible for a small proportion of PPROM cases. However, a non-infectious mechanism is likely responsible for the remainder. Therefore, patient-specific selection of targeted antibiotic therapy may improve efficacy and patient outcomes.

Moreover, vaginal dysbiosis just prior to delivery was strongly associated with both chorioamnionitis with funisitis and maternal serum markers of infection and inflammation. Cross-sectional and longitudinal analyses showed that erythromycin failed to resolve this dysbiosis within 1 week of treatment, which coincides with delivery in approximately 80% of cases, and instead was associated with a significant and persistent increase in dysbiotic community structures. This increase was particularly apparent in women with initial colonisation of *Lactobacillus* species. In women with pre-existing dysbiosis, erythromycin was associated with a reduction in species richness and diversity. However, communities continued to be depleted of *Lactobacillus* species, indicating there was a restructuring of the highly diverse compositions. Erythromycin treatment beyond 1 week was associated with a recovery of pre-treatment levels of *Lactobacillus* species dominance. However, the proportion of dysbiotic communities remained unchanged throughout the treatment course.

The use of erythromycin treatment for PPROM is largely driven by results from the ORACLE I trial, which reported prolongation of pregnancy for 48 hours (34.8% vs. 40.7%, *P* = 0.004), reduced need for supplemental oxygen (31.1% vs. 35.6%, *P* = 0.02) and a 2.2% reduction in composite neonatal morbidity (neonatal death, chronic lung disease or major cerebral abnormality; 11.2% vs. 14.4%, *P* = 0.02) in women randomised to orally administered erythromycin prophylaxis compared to placebo [67]. The trial also included randomisation arms of co-amoxiclav and co-amoxiclav plus erythromycin, which were both associated with a significant increase in the risk of necrotising enterocolitis (1.9% vs. 0.5%, *P* = 0.001 and 1.7% vs. 0.5%, *P* = 0.005, respectively). The decision to test these antibiotics in the trial was based upon their broad spectrum, complementary ranges of activities, comparatively minimal contraindications in pregnancy and the opportunity to test a macrolide and β-lactam antibiotic. Beneficial outcomes associated with erythromycin treatment in PPROM are often attributed to its assumed inhibition of ascending vaginal infection, but this seems unlikely considering erythromycin concentration in the vaginal lumen following oral dosing is low [68], reaching a mean inhibitory concentration effective against *Lactobacillus* species [44, 69, 70], but not against most other species known to colonise the vagina [44]. This provides a possible explanation for the reduction of *Lactobacillus* spp. and increased diversity and richness of bacterial communities observed in our study following erythromycin treatment that occurred without a reduction in overall bacterial load.

Despite adverse effects on vaginal microbiota composition, reported improvements in neonatal and maternal outcomes following erythromycin treatment for PPROM may be attributable to anti-bacterial activity at other gestational tissue sites (e.g. the placenta) or to other modes of action. Erythromycin is used primarily as an anti-inflammatory for the treatment of chronic inflammatory lung disease (panbronchiolitis) [71, 72] and has been shown to have tocolytic action in vitro [73]. Considering that the placental transfer of erythromycin into the fetal circulation is low (approximately 2%) [74], neonatal benefits are more likely due to action on maternal tissues and subsequent inhibition of inflammatory mediators that could cross the placenta. Nevertheless, vaginal delivery acts as a high-dose inoculum to the neonate, which shapes the composition of the early infant gut microbiome [25, 75–78], which is in turn linked to short-term and long-term health outcomes [79, 80]. Therefore, aberrant augmentation of vaginal bacterial communities towards dysbiosis just prior to delivery is undesirable and may contribute to poor neonatal outcomes. In our study, vaginal dysbiosis and enrichment of *Sneathia* spp. and other potential pathogenic bacteria (e.g. *Streptococcus agalactiae*) just prior to delivery were observed in cases subsequently developing EONS. *Sneathia* spp. are often associated with bacterial vaginosis [81] and their colonisation of the vagina has been linked to various adverse pregnancy outcomes including septic abortion [82], neonatal bacteraemia [83], neonatal meningitis [84] and chorioamnionitis [85]. Vaginal bacterial communities isolated from cases subsequently developing EONS were almost entirely void of *Lactobacillus crispatus*. Colonisation of *L. crispatus* is highly stable in healthy pregnancies from similar cohorts to those studied here [22, 25] and dominant colonisation in early pregnancy is associated with protection against preterm birth in those women at high risk [25]. Our data indicate that *L. crispatus* may also provide protection against subsequent development of EONS.

Although our study size is limited by the practicalities and costs associated with prospectively recruiting large numbers of women subsequently experiencing PPROM, it represents a unique assessment of vaginal microbiota prior to rupture of fetal membranes and is the largest study of the vaginal microbiota in the context of PPROM to date. Given the observational nature of the study, it was not possible, in the context of UK National Health Service care, to longitudinally sample a cohort of women following PPROM who did not receive erythromycin as part of treatment guidelines issued by the WHO, National Institute for Health and Care Excellence (NICE), the Royal College of Obstetricians and Gynaecologists and the recruiting hospital. Therefore, it is difficult to separate the potential temporal impact of membrane rupture on shaping vaginal community structure from the pharmacological effect of erythromycin. Amniotic fluid is highly alkaline with a pH of 7.1–7.3 and contains antimicrobial peptides [86] that may account for the reduction in bacterial load following rupture observed in our study, prior to erythromycin treatment. However, even if erythromycin is not the primary driver of dysbiosis, our data show that it fails to improve the composition of the vaginal microbiome by eradicating potential pathogens or reducing overall bacterial load and is detrimental for individuals with a *Lactobacillus* spp. dominated microbiome.

## Conclusions

Our data show that the composition of the vaginal microbiome is a risk factor for subsequent PPROM and is associated with adverse short-term maternal and neonatal outcomes. Erythromycin prophylaxis promotes *Lactobacillus* spp. depletion and increased the bacterial diversity of the microbiota, which correlates with increased prevalence of chorioamnionitis, funisitis and EONS. There is an urgent need to review the continued use of prophylactic erythromycin in the context of PPROM and to develop alternative treatment strategies, such as patient-specific therapy, selective antibiotics or different routes of administration, to resolve the vaginal dysbiosis associated with PPROM and improve maternal and neonatal outcomes.

## Additional file

Additional file 1: Figure S1. Characteristics of vaginal microbiota groups (VMGs) defined using Ward clustering. **Figure S2**. Bacterial taxonomic groups discriminate between normal-term delivery and samples taken following membrane rupture. **Figure S3**. Bacterial taxonomic groups associated with early onset neonatal sepsis (EONS) following PPROM, for neonates delivered at or before 28 weeks gestation (*n* = 27). **Table S1**. Bacterial diversity, richness and relative abundance of *Lactobacillus* spp. for VMGs 1–8. **Table S2**. Linear regression analysis comparing proportion of *Lactobacillus* spp. dominance across all patient groups corrected for potential confounders. **Table S3**. Linear regression analysis comparing proportion of *Lactobacillus* spp. dominance in paired samples before and after 48 hours erythromycin treatment. **Table S4**. Maternal and neonatal factors in the presence and absence of chorioamnionitis +/- funisitis. **Table S5**. Linear regression analysis comparing proportion of *Lactobacillus* spp. dominance in cases with and without chorioamnionitis +/- funisitis. **Table S6**. Maternal and neonatal factors associated with EONS. (DOCX 1632 kb)

## Abbreviations

ANOVA: Analysis of variance
BMI: Body mass index
BW: Birth weight
CRP: C-reactive protein
DNA: Deoxyribonucleic acid
EONS: Early onset neonatal sepsis
GA: Gestational age
HIV: human immunodeficiency virus
LDA: Latent discriminatory analysis
LEfSe: Linear discriminant analysis with effect size
MR: Membrane Rupture
NICE: National Institute for Health and Care Excellence
PCR: Polymerase chain reaction
PPROM: Preterm prelabour rupture of the fetal membranes
RDP: Ribosomal Database Project
STAMP: Statistical Analysis of Metagenomic Profiles
VMG: Vaginal microbiota group
WCC: White cell count
WHO: World Health Organization

## References

[1] Liu L, Oza S, Hogan D, Perin J, Rudan I, Lawn JE (2015). Global, regional, and national causes of child mortality in 2000–13, with projections to inform post-2015 priorities: an updated systematic analysis. Lancet.

[2] Iacovidou N, Varsami M, Syggellou A (2010). Neonatal outcome of preterm delivery. Ann NY Acad Sci..

[3] Parry S, Strauss JF (1998). Premature rupture of the fetal membranes. N Engl J Med.

[4] Lamont RF, Duncan SLB, Mandal D, Bassett P (2003). Intravaginal clindamycin to reduce preterm birth in women with abnormal genital tract flora. Obstet Gynecol.

[5] Pappas A, Kendrick DE, Shankaran S (2014). Chorioamnionitis and early childhood outcomes among extremely low-gestational-age neonates. JAMA Pediatr.

[6] Chandiramani M, Bennett PR, Brown R, Lee Y, MacIntyre DA (2014). Vaginal microbiome-pregnant host interactions determine a significant proportion of preterm labour. Fetal Matern Med Rev.

[7] Kanayama N, Terao T, Horiuchi K (1988). The role of human neutrophil elastase in the premature rupture of membranes. Asia Oceania J Obstet Gynaecol.

[8] Fortunato SJ, Menon R, Lombardi SJ (2002). Role of tumor necrosis factor-α in the premature rupture of membranes and preterm labor pathways. Am J Obstet Gynecol.

[9] Shobokshi A, Shaarawy M (2002). Maternal serum and amniotic fluid cytokines in patients with preterm premature rupture of membranes with and without intrauterine infection. Int J Gynaecol Obstet.

[10] Helmig BR, Romero R, Espinoza J, Chaiworapongsa T, Bujold E, Gomez R (2002). Neutrophil elastase and secretory leukocyte protease inhibitor in prelabor rupture of membranes, parturition and intra-amniotic infection. J Matern Fetal Neonatal Med.

[11] Fortner KB, Grotegut CA, Ransom CE, Bentley RC, Feng L, Lan L (2014). Bacteria localization and chorion thinning among preterm premature rupture of membranes. PloS One.

[12] Peaceman AM, Lai Y, Rouse DJ, Spong CY, Mercer BM, Varner MW (2015). Length of latency with preterm premature rupture of membranes before 32 weeks' gestation. Am J Perinatol.

[13] Rocha G, Proenca E, Quintas C, Rodrigues T, Guimaraes H (2007). Chorioamnionitis and brain damage in the preterm newborn. J Matern Fetal Neonatal Med.

[14] Lu H, Wang Q, Lu J, Zhang Q, Kumar P (2016). Risk factors for intraventricular hemorrhage in preterm infants born at 34 weeks of gestation or less following preterm premature rupture of membranes. J Stroke Cerebrovasc Dis.

[15] Vigneswaran R (2000). Infection and preterm birth: evidence of a common causal relationship with bronchopulmonary dysplasia and cerebral palsy. J Paediatr Child Health.

[16] Yoon BH, Romero R, Park JS, Kim CJ, Kim SH, Choi JH (2000). Fetal exposure to an intra-amniotic inflammation and the development of cerebral palsy at the age of three years. Am J Obstet Gynecol.

[17] Drassinower D, Friedman AM, Obican SG, Levin H, Gyamfi-Bannerman C. Prolonged latency of preterm premature rupture of membranes and risk of cerebral palsy. J Matern Fetal Neonatal Med. 2016;29(17):2748–52. 10.3109/14767058.2015.1107539 26595801

[18] van Dillen J, Zwart J, Schutte J, van Roosmalen J (2010). Maternal sepsis: epidemiology, etiology and outcome. Curr Opin Infect Dis.

[19] Puri K, Taft DH, Ambalavanan N, Schibler KR, Morrow AL, Kallapur SG (2016). Association of chorioamnionitis with aberrant neonatal gut colonization and adverse clinical outcomes. PloS One.

[20] Gajer P, Brotman RM, Bai G, Sakamoto J, Schutte UM, Zhong X (2012). Temporal dynamics of the human vaginal microbiota. Sci Transl Med.

[21] Aagaard K, Riehle K, Ma J, Segata N, Mistretta TA, Coarfa C (2012). A metagenomic approach to characterization of the vaginal microbiome signature in pregnancy. PloS One.

[22] MacIntyre DA, Chandiramani M, Lee YS, Kindinger L, Smith A, Angelopoulos N (2015). The vaginal microbiome during pregnancy and the postpartum period in a European population. Sci Rep..

[23] Romero R, Hassan SS, Gajer P, Tarca AL, Fadrosh DW, Nikita L (2014). The composition and stability of the vaginal microbiota of normal pregnant women is different from that of non-pregnant women. Microbiome..

[24] Reid G, Younes JA, Van der Mei HC, Gloor GB, Knight R, Busscher HJ (2011). Microbiota restoration: natural and supplemented recovery of human microbial communities. Nat Rev Microbiol.

[25] Kindinger LM, Bennett PR, Lee YS, Marchesi JR, Smith A, Cacciatore S (2017). The interaction between vaginal microbiota, cervical length, and vaginal progesterone treatment for preterm birth risk. Microbiome.

[26] Petricevic L, Domig KJ, Nierscher FJ, Sandhofer MJ, Fidesser M, Krondorfer I (2014). Characterisation of the vaginal *Lactobacillus* microbiota associated with preterm delivery. Sci Rep..

[27] McGregor JA, French JI, Seo K (1993). Premature rupture of membranes and bacterial vaginosis. Am J Obstet Gynecol.

[28] Flynn CA, Helwig AL, Meurer LN (1999). Bacterial vaginosis in pregnancy and the risk of prematurity: a meta-analysis. J Fam Pract.

[29] Hillier SL, Nugent RP, Eschenbach DA, Krohn MA, Gibbs RS, Martin DH (1995). Association between bacterial vaginosis and preterm delivery of a low-birth-weight infant. N Engl J Med.

[30] DiGiulio DB, Callahan BJ, McMurdie PJ, Costello EK, Lyell DJ, Robaczewska A (2015). Temporal and spatial variation of the human microbiota during pregnancy. Proc Natl Acad Sci USA.

[31] Kindinger LM, MacIntyre DA, Lee YS, Marchesi JR, Smith A, McDonald JA (2016). Relationship between vaginal microbial dysbiosis, inflammation, and pregnancy outcomes in cervical cerclage. Sci Transl Med.

[32] Gibbs RS (1993). Chorioamnionitis and bacterial vaginosis. Am J Obstet Gynecol.

[33] Martius J, Eschenbach DA (1990). The role of bacterial vaginosis as a cause of amniotic fluid infection, chorioamnionitis and prematurity – a review. Arch Gynecol Obstet.

[34] Takei H, Ruiz B (2006). Shift in vaginal flora (bacterial vaginosis) and the frequency of chorioamnionitis in a high-risk population. Acta Cytol.

[35] Baldwin EA, Walther-Antonio M, MacLean AM, Gohl DM, Beckman KB, Chen J (2015). Persistent microbial dysbiosis in preterm premature rupture of membranes from onset until delivery. PeerJ..

[36] Kacerovsky M, Vrbacky F, Kutova R, Pliskova L, Andrys C, Musilova I (2015). Cervical microbiota in women with preterm prelabor rupture of membranes. PloS One.

[37] Paramel Jayaprakash T, Wagner EC, van Schalkwyk J, Albert AY, Hill JE, Money DM (2016). High diversity and variability in the vaginal microbiome in women following preterm premature rupture of membranes (PPROM): a prospective cohort study. PloS One.

[38] Ramsey PS, Nuthalapaty FS, Lu G, Ramin S, Nuthalapaty ES, Ramin KD (2004). Contemporary management of preterm premature rupture of membranes (PPROM): a survey of maternal-fetal medicine providers. Am J Obstet Gynecol.

[39] Kenyon S, Pike K, Jones D, Brocklehurst P, Marlow N, Salt A (2010). Has publication of the results of the ORACLE Children Study changed practice in the UK?. BJOG.

[40] NICE. Preterm labour and birth. 2015. http://www.nice.org.uk/guidance/ng25.

[41] Kenyon S, Taylor DJ, Tarnow-Mordi WO (2002). ORACLE – antibiotics for preterm prelabour rupture of the membranes: short-term and long-term outcomes. Acta Paediatr Suppl.

[42] Kenyon S, Pike K, Jones DR, Brocklehurst P, Marlow N, Salt A (2008). Childhood outcomes after prescription of antibiotics to pregnant women with preterm rupture of the membranes: 7-year follow-up of the ORACLE I trial. Lancet.

[43] Marlow N, Bower H, Jones D, Brocklehurst P, Kenyon S, Pike K, et al. The ORACLE Children Study: educational outcomes at 11 years of age following antenatal prescription of erythromycin or co-amoxiclav. Arch Dis Child Fetal Neonatal Ed. 2016;102(2):F131-F135. 10.1136/archdischild-2015-310144 PMC5339554 27515985

[44] Kuriyama T, Williams DW, Yanagisawa M, Iwahara K, Shimizu C, Nakagawa K (2007). Antimicrobial susceptibility of 800 anaerobic isolates from patients with dentoalveolar infection to 13 oral antibiotics. Oral Microbiol Immunol.

[45] Lee MY, Kim MH, Lee WI, Kang SY, Jeon YL (2016). Prevalence and antibiotic susceptibility of *Mycoplasma hominis* and *Ureaplasma urealyticum* in pregnant women. Yonsei Med J.

[46] Desjardins M, Delgaty KL, Ramotar K, Seetaram C, Toye B (2004). Prevalence and mechanisms of erythromycin resistance in group A and group B *Streptococcus*: implications for reporting susceptibility results. J Clin Microbiol.

[47] Meeraus WH, Petersen I, Gilbert R (2015). Association between antibiotic prescribing in pregnancy and cerebral palsy or epilepsy in children born at term: a cohort study using the health improvement network. PloS One.

[48] Korpela K, Salonen A, Virta LJ, Kekkonen RA, Forslund K, Bork P (2016). Intestinal microbiome is related to lifetime antibiotic use in Finnish pre-school children. Nat Commun..

[49] Sundquist A, Bigdeli S, Jalili R, Druzin ML, Waller S, Pullen KM (2007). Bacterial flora-typing with targeted, chip-based pyrosequencing. BMC Microbiol..

[50] Kozich JJ, Westcott SL, Baxter NT, Highlander SK, Schloss PD (2013). Development of a dual-index sequencing strategy and curation pipeline for analyzing amplicon sequence data on the MiSeq Illumina sequencing platform. Appl Environ Microbiol.

[51] Wang Q, Garrity GM, Tiedje JM, Cole JR (2007). Naive Bayesian classifier for rapid assignment of rRNA sequences into the new bacterial taxonomy. Appl Environ Microbiol.

[52] Edgar RC (2010). Search and clustering orders of magnitude faster than BLAST. Bioinformatics.

[53] Liu CM, Aziz M, Kachur S, Hsueh P-R, Huang Y-T, Keim P (2012). BactQuant: an enhanced broad-coverage bacterial quantitative real-time PCR assay. BMC Microbiol.

[54] Parks DH, Beiko RG (2010). Identifying biologically relevant differences between metagenomic communities. Bioinformatics.

[55] Segata N, Izard J, Waldron L, Gevers D, Miropolsky L, Garrett WS (2011). Metagenomic biomarker discovery and explanation. Genome Biol.

[56] Ravel J, Gajer P, Abdo Z, Schneider GM, Koenig SS, McCulle SL (2011). Vaginal microbiome of reproductive-age women. Proc Natl Acad Sci USA.

[57] Borgdorff H, Tsivtsivadze E, Verhelst R, Marzorati M, Jurriaans S, Ndayisaba GF (2014). *Lactobacillus*-dominated cervicovaginal microbiota associated with reduced HIV/STI prevalence and genital HIV viral load in African women. ISME J.

[58] Kenyon S, Boulvain M, Neilson JP (2013). Antibiotics for preterm rupture of membranes. Cochrane Database Syst Rev..

[59] Cousens S, Blencowe H, Gravett M, Lawn JE (2010). Antibiotics for pre-term pre-labour rupture of membranes: prevention of neonatal deaths due to complications of pre-term birth and infection. Int J Epidemiol..

[60] World Health Organization. WHO recommendations on interventions to improve preterm birth outcomes. 2015. http://www.who.int/reproductivehealth/publications/maternal_perinatal_health/preterm-birth-guideline. 26447264

[61] Yudin MH, van Schalkwyk J, Van Eyk N, Boucher M, Castillo E, Cormier B (2009). Antibiotic therapy in preterm premature rupture of the membranes. J Obstet Gynaecol Can.

[62] Seelbach-Goebel B (2013). Antibiotic therapy for premature rupture of membranes and preterm labor and effect on fetal outcome. Geburtshilfe Frauenheilkd.

[63] Royal Australian and New Zealand College of Obstetricians and Gynaecologists. Green-top Guideline No. 44. Preterm prelabour rupture of membranes. 2010. https://www.ranzcog.edu.au/RANZCOG_SITE/media/RANZCOGMEDIA/Women%27s%20Health/Statement%20and%20guidelines/Clinical-Obstetrics/RCOG-Preterm-Prelabour-Rupture-of-Membranes.pdf?ext=.pdf.

[64] Goldenberg RL, Culhane JF, Iams JD, Romero R (2008). Epidemiology and causes of preterm birth. Lancet.

[65] Romero R, Dey SK, Fisher SJ (2014). Preterm labor: one syndrome, many causes. Science.

[66] Romero R, Espinoza J, Goncalves LF, Kusanovic JP, Friel L, Hassan S (2007). The role of inflammation and infection in preterm birth. Semin Reprod Med.

[67] Kenyon SL, Taylor DJ, Tarnow-Mordi W (2001). Broad-spectrum antibiotics for preterm, prelabour rupture of fetal membranes: the ORACLE I randomised trial. Lancet.

[68] Iliopoulou A, Thin RN, Turner P (1981). Fluorimetric and microbiological assays of erythromycin concentrations in plasma and vaginal washings. Br J Vener Dis.

[69] Harwich MD, Serrano MG, Fettweis JM, Alves JM, Reimers MA, Buck GA (2012). Genomic sequence analysis and characterization of *Sneathia amnii* sp. nov. BMC Genomics.

[70] Salminen MK, Rautelin H, Tynkkynen S, Poussa T, Saxelin M, Valtonen V (2006). *Lactobacillus* bacteremia, species identification, and antimicrobial susceptibility of 85 blood isolates. Clin Infect Dis.

[71] Koyama H, Geddes DM (1997). Erythromycin and diffuse panbronchiolitis. Thorax.

[72] Desaki M, Okazaki H, Sunazuka T, Omura S, Yamamoto K, Takizawa H (2004). Molecular mechanisms of anti-inflammatory action of erythromycin in human bronchial epithelial cells: possible role in the signaling pathway that regulates nuclear factor-κB activation. Antimicrob Agents Chemother.

[73] Celik H, Ayar A, Sapmaz E (2001). Effects of erythromycin on stretch-induced contractile activity of isolated myometrium from pregnant women. Acta Obstet Gynecol Scand.

[74] Bulska M, Szczesniak P, Pieta-Dolinska A, Oszukowski P, Orszulak-Michalak D (2015). The placental transfer of erythromycin in human pregnancies with group B streptococcal infection. Ginekologia polska.

[75] Dominguez-Bello MG, Costello EK, Contreras M, Magris M, Hidalgo G, Fierer N (2010). Delivery mode shapes the acquisition and structure of the initial microbiota across multiple body habitats in newborns. Proc Natl Acad Sci USA.

[76] Dominguez-Bello MG, De Jesus-Laboy KM, Shen N, Cox LM, Amir A, Gonzalez A (2016). Partial restoration of the microbiota of cesarean-born infants via vaginal microbial transfer. Nat Med.

[77] Yassour M, Vatanen T, Siljander H, Hamalainen AM, Harkonen T, Ryhanen SJ (2016). Natural history of the infant gut microbiome and impact of antibiotic treatment on bacterial strain diversity and stability. Sci Transl Med.

[78] Bokulich NA, Chung J, Battaglia T, Henderson N, Jay M, Li H (2016). Antibiotics, birth mode, and diet shape microbiome maturation during early life. Sci Transl Med.

[79] Fujimura KE, Sitarik AR, Havstad S, Lin DL, Levan S, Fadrosh D (2016). Neonatal gut microbiota associates with childhood multisensitized atopy and T cell differentiation. Nat Med.

[80] Jasarevic E, Howerton CL, Howard CD, Bale TL (2015). Alterations in the vaginal microbiome by maternal stress are associated with metabolic reprogramming of the offspring gut and brain. Endocrinology.

[81] Koumans EH, Sternberg M, Bruce C, McQuillan G, Kendrick J, Sutton M (2007). The prevalence of bacterial vaginosis in the United States, 2001–2004; associations with symptoms, sexual behaviors, and reproductive health. Sex Transm Dis.

[82] Boennelycke M, Christensen JJ, Arpi M, Krause S (2007). *Leptotrichia amnionii* found in septic abortion in Denmark. Scand J Infect Dis.

[83] Hanff PA, Rosol-Donoghue JA, Spiegel CA, Wilson KH, Moore LH (1995). *Leptotrichia sanguinegens* sp. nov., a new agent of postpartum and neonatal bacteremia. Clin Infect Dis.

[84] Devi U, Bora R, Das JK, Malik V, Mahanta J (2014). Sneathia species in a case of neonatal meningitis from Northeast India. Oxf Med Case Rep.

[85] Han YW, Shen T, Chung P, Buhimschi IA, Buhimschi CS (2009). Uncultivated bacteria as etiologic agents of intra-amniotic inflammation leading to preterm birth. J Clin Microbiol.

[86] Underwood MA, Gilbert WM, Sherman MP (2005). Amniotic fluid: not just fetal urine anymore. J Perinatol.

