# Supplementary material for: Vaginal dysbiosis increases risk of preterm fetal membrane rupture, neonatal sepsis and is exacerbated by erythromycin
Source: BMC Med. 2018 Jan 24;16:9. doi: 10.1186/s12916-017-0999-x (PMC5782380; doi:10.1186/s12916-017-0999-x)
Supplement: Additional file 1: Figure S1. — Characteristics of vaginal microbiota groups (VMGs) defined using Ward clustering. Figure S2. Bacterial taxonomic groups discriminate between normal-term delivery and samples taken following membrane rupture. Figure S3. Bacterial taxonomic groups associated with early onset neonatal sepsis (EONS) following PPROM, for neonates delivered at or before 28 weeks gestation (n = 27). Table S1. Bacterial diversity, richness and relative abundance of Lactobacillus spp. for VMGs 1–8. Table S2. Linear regression analysis comparing proportion of Lactobacillus spp. dominance across all patient groups corrected for potential confounders. Table S3. Linear regression analysis comparing proportion of Lactobacillus spp. dominance in paired samples before and after 48 hours erythromycin treatment. Table S4. Maternal and neonatal factors in the presence and absence of chorioamnionitis +/- funisitis. Table S5. Linear regression analysis comparing proportion of Lactobacillus spp. dominance in cases with and without chorioamnionitis +/- funisitis. Table S6. Maternal and neonatal factors associated with EONS. (DOCX 1632 kb) [file 12916_2017_999_MOESM1_ESM.docx]

**Additional Files**

**Additional Figures**


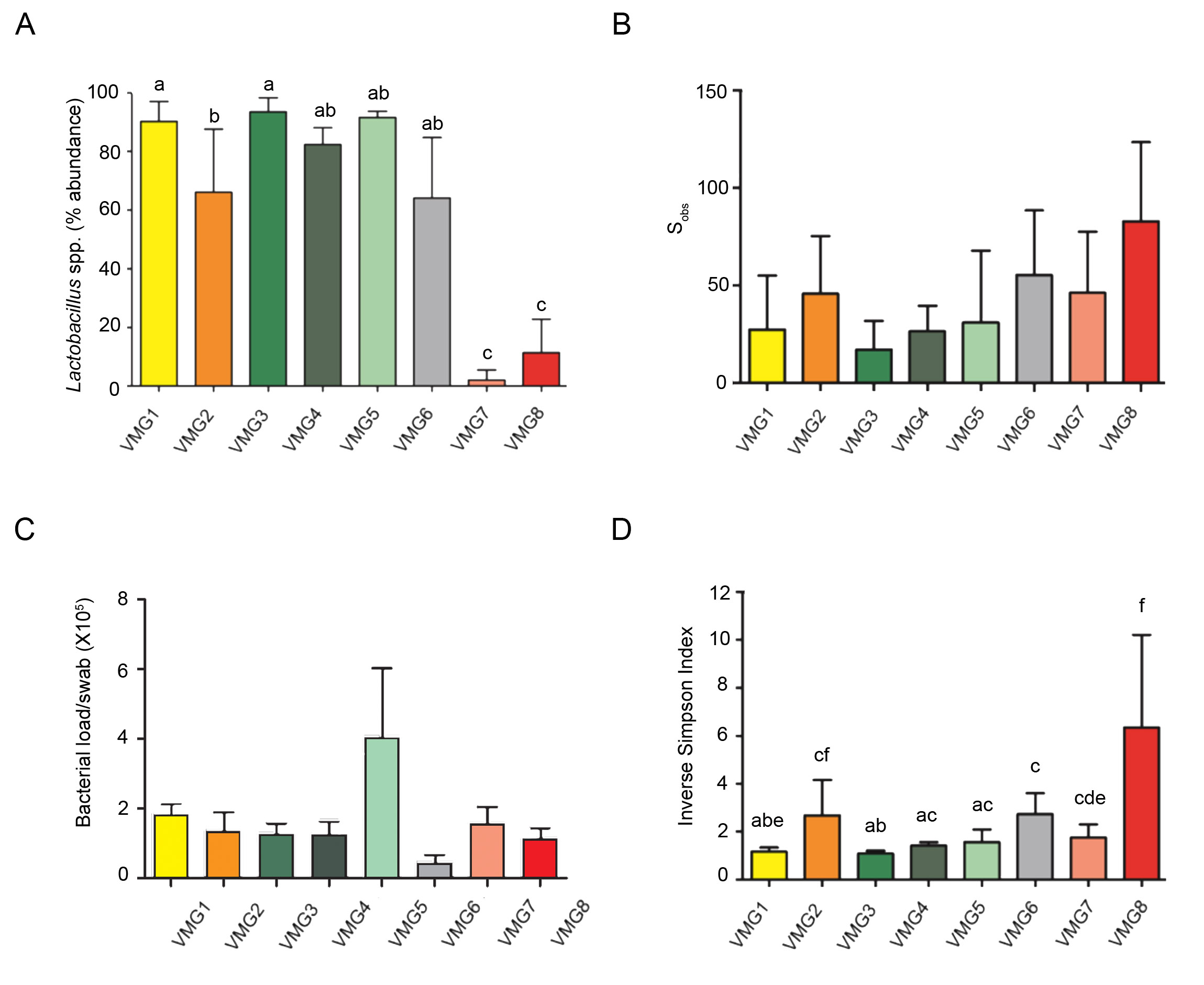


**Figure S1. Characteristics of Vaginal Microbiota Groups defined using Ward clustering**. A total of 8 vaginal microbiota groups (VMG) were identified. **(A)** VMG 1,3,4 and 5 are dominated by *Lactobacillus*, VMG 2 and 6 have intermediate *Lactobacillus* abundance and VMG 7 and 8 have significantly lower *Lactobacillus* abundance (*P* < 0.0001, Kruskal Wallis) **(B)** Species observed were lowest in the *Lactobacillus* dominant groups (VMG 1,3,4 and 5) and highest in groups 2,6 and 8. **(C)** Bacterial load was comparable across all groups **(D)** Inverse simpson scores were significantly higher in VMG 2,6 and 8. These differential characteristics were used to sub-group the Vaginal Microbiota Groups into *Lactobacillus* dominant (1,3,4,5) Intermediate *(*2,6*)* and deplete (7,8) communities. Letters indicate significant differences detected following post-hoc, pair-wise comparison (Dunn’s multiple comparison, *P* < 0.05). Shared letters indicate no difference; a lack of shared letters indicates a significant difference in medians between the groups.


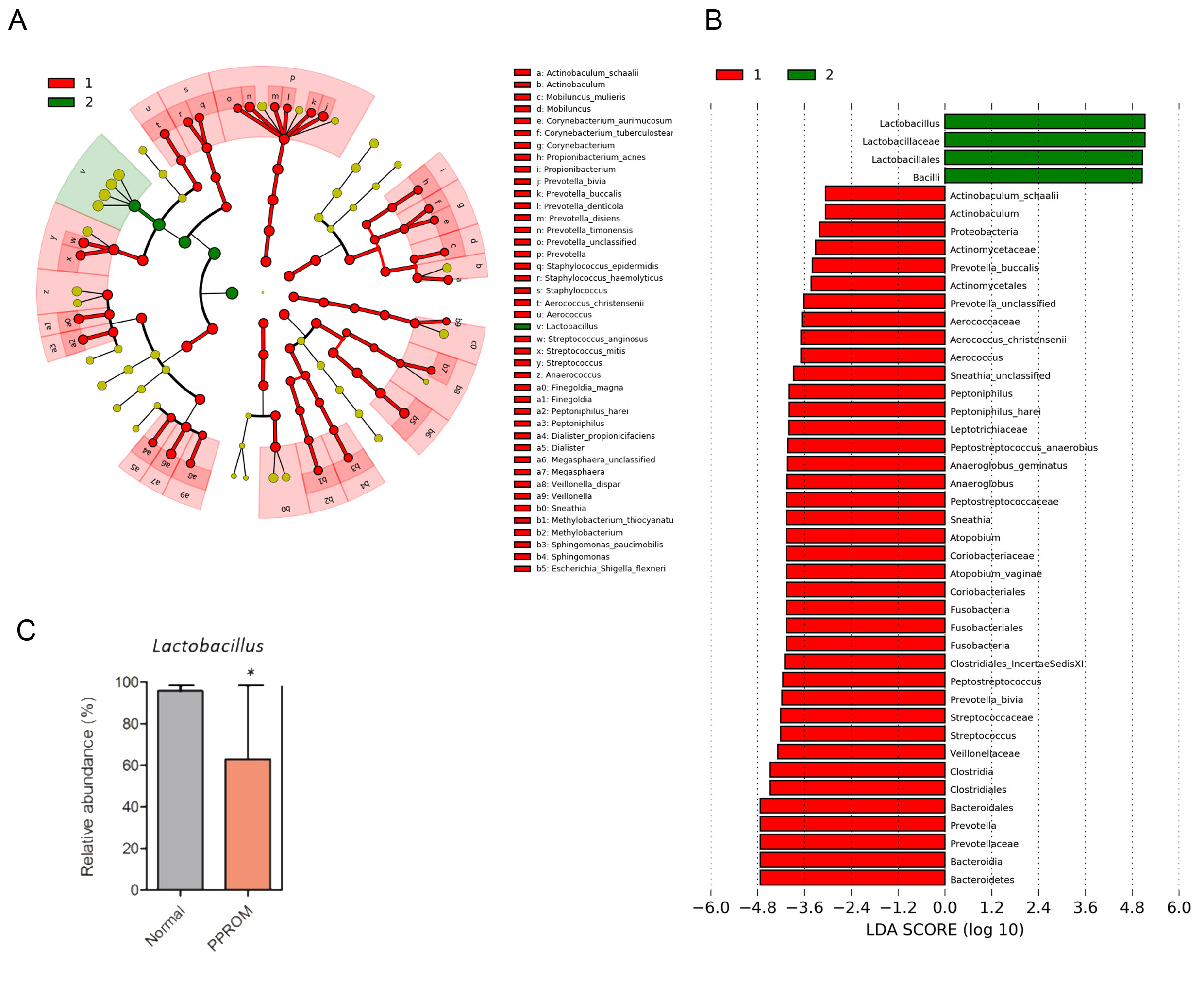


**Figure S2. Bacterial taxonomic groups discriminate between normal term delivery and samples taken following membrane rupture. (A)** Differentially abundant microbial clades and nodes identified between gestational age matched samples from normal pregnancies delivering at term and samples taken following PPROM prior to erythromycin treatment identified by LEfSe and presented as a cladogram. **(B)** LDA with effect size for each differentially abundant species, normal pregnancy is characterised by *Lactobacillus* spp. An expansion in diversity following membrane rupture including increased levels of *Streptococcus* spp., *Sneathia* spp. and *Prevotella* spp. **(C)** There is a reduction in Lactobacillus abundance following membrane rupture (*P* = 0.039, Mann Whitney).

**
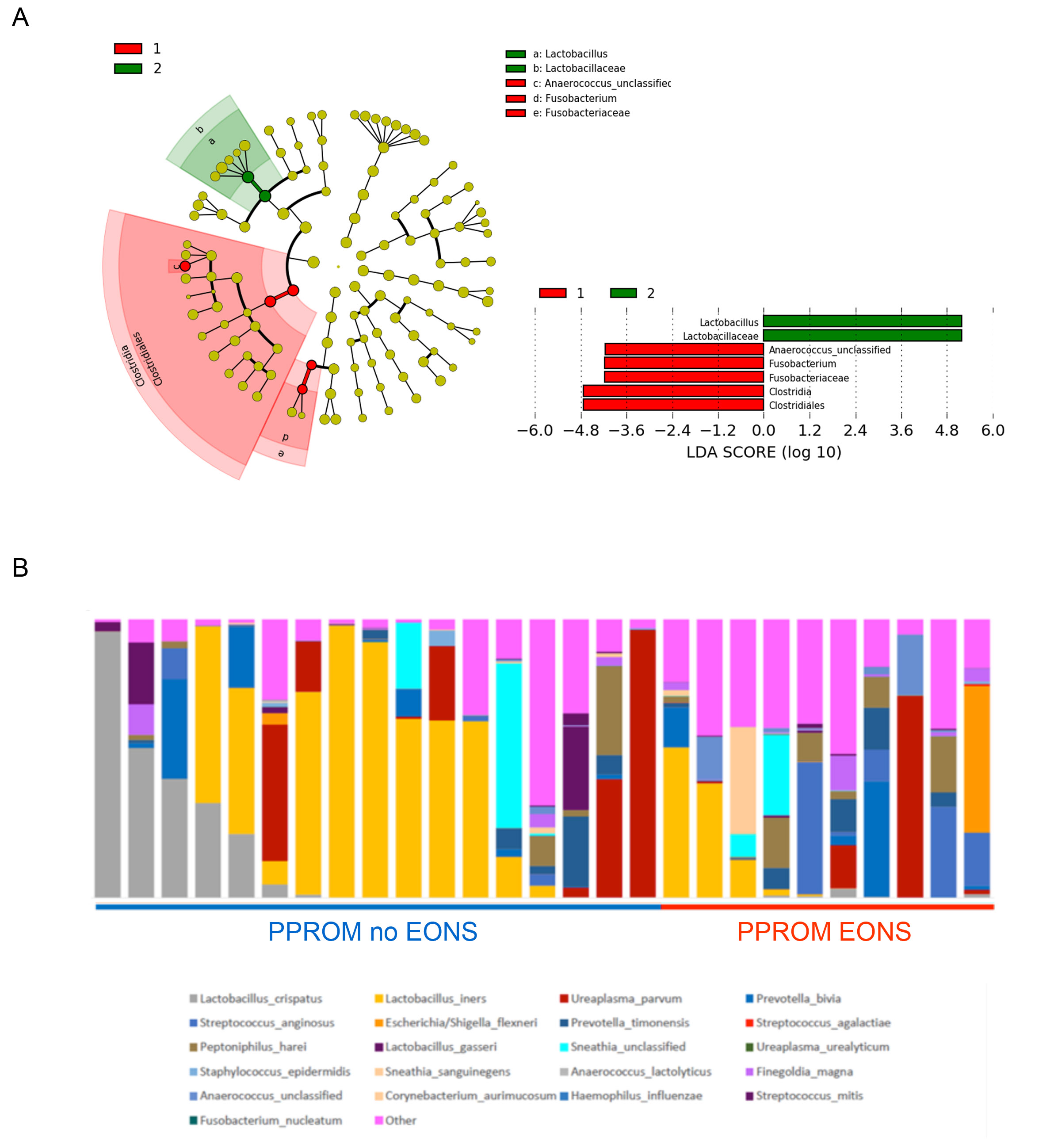
**

**Figure S3. Bacterial taxonomic groups associated with Early Onset Neonatal Sepsis (EONS) following PPROM, for neonates delivered at or before 28 weeks gestation (n=27). (A)** Differentially abundant microbial clades and nodes identified from the last vaginal sample taken prior to delivery according to the presence of neonatal sepsis identified using LefSe analysis and presented as a cladogram **(B)** LDA to estimate the effect size for each differentially abundant taxa. *Lactobacillus* spp. were differentially expressed amongst women whose babies did not develop EONS whilst vaginal *Fusobacterium* was associated with EONS.

**Additional Tables**

**Table S1. Bacterial diversity, richness and *Lactobacillu*s spp. relative abundance of Vaginal Microbiota Groups 1-8**

| VMG | Sobs | Inv. Simpson Index | Shannon Index | Dominant *Lactobacillus* spp. | Average Abundance *Lactobacillus* spp. (%) |
| --- | --- | --- | --- | --- | --- |
| 1 | 15 (9-42) | 1.10 (1.01-1.26) | 0.29 (0.03-0.48) | *L.iners* | 92.3 (84.9-04.9) |
| 2 | 36 (26-54) | 2.08 (1.83-3.10) | 1.13 (0.90-1.56) | *L.iners* | 68.3 (49.8-81.1) |
| 3 | 11 (6.-26) | 1.03 (1.01- 1.10) | 0.11 (0.03-0.25) | *L.crispatus* | 93.3 (92.2-98.0) |
| 4 | 26 (14-40) | 1.42 (1.29-1.54) | 0.63 (0.5-0.65) | *L.gasseri* | 80.3 (78.1-87.7) |
| 5 | 10 (7-77) | 1.56 (1.00-2.04) | 0.71 (0.02-0.92) | *L.jensenii* | 92.6 (89.5-93.1) |
| 6 | 38 (32-87) | 2.85 (1.82-3.45) | 1.4 (0.81-1.90) | *L.crispatus* | 62.3 (44.6-87.9) |
| 7 | 42 (18-69) | 1.74 (1.20-2.30) | 0.91 (0.53-1.27) | NA | 0.41 (0.13-2.76) |
| 8 | 85 (46-117) | 5.00 (3.70-7.40) | 2.28 (1.73-2.74) | NA | 9.3 (0.8-16.1) |

**Table S2. Linear regression analysis comparing proportion of *Lactobacillus* spp dominance across all patient groups corrected for potential confounders as described in Methods.**

| Patient  Groups | *Lactobacillus* Spp. dominance | estimate | se | t | p | q | n | paired |
| --- | --- | --- | --- | --- | --- | --- | --- | --- |
| 1vs2 | 1 | 0.569 | 0.204 | 2.789 | 5.3E-03 | 2.2E-02 | 35 | 0 |
| 1vs2 | 2 | -0.214 | 0.142 | -1.505 | 1.3E-01 | 2.8E-01 | 35 | 0 |
| 1vs2 | 3 | -0.355 | 0.162 | -2.190 | 2.9E-02 | 8.6E-02 | 35 | 0 |
| 1vs3 | 1 | 0.484 | 0.131 | 3.686 | 2.3E-04 | 2.4E-03 | 59 | 0 |
| 1vs3 | 2 | -0.142 | 0.090 | -1.565 | 1.2E-01 | 2.7E-01 | 59 | 0 |
| 1vs3 | 3 | -0.342 | 0.111 | -3.081 | 2.1E-03 | 1.4E-02 | 59 | 0 |
| 2vs3 | 1 | 0.000 | 0.000 | 0.000 | 1.0E+00 | 1.0E+00 | 54 | 1 |
| 2vs3 | 2 | 0.000 | 0.000 | 0.000 | 1.0E+00 | 1.0E+00 | 54 | 1 |
| 2vs3 | 3 | 0.000 | 0.000 | 0.000 | 1.0E+00 | 1.0E+00 | 54 | 1 |
| 3vs4 | 1 | 0.361 | 0.084 | 4.291 | 1.8E-05 | 3.7E-04 | 82 | 1 |
| 3vs4 | 2 | -0.229 | 0.091 | -2.507 | 1.2E-02 | 4.3E-02 | 82 | 1 |
| 3vs4 | 3 | -0.129 | 0.100 | -1.285 | 2.0E-01 | 3.8E-01 | 82 | 1 |
| 4vs5 | 1 | 0.000 | 0.000 | 0.000 | 1.0E+00 | 1.0E+00 | 65 | 1 |
| 4vs5 | 2 | 0.000 | 0.000 | 0.000 | 1.0E+00 | 1.0E+00 | 65 | 1 |
| 4vs5 | 3 | 0.000 | 0.000 | 0.000 | 1.0E+00 | 1.0E+00 | 65 | 1 |
| 4vs6 | 1 | -0.237 | 0.085 | -2.803 | 5.1E-03 | 2.2E-02 | 69 | 1 |
| 4vs6 | 2 | 0.000 | 0.000 | 0.000 | 1.0E+00 | 1.0E+00 | 69 | 1 |
| 4vs6 | 3 | 0.086 | 0.094 | 0.920 | 3.6E-01 | 6.3E-01 | 69 | 1 |
| 5vs6 | 1 | 0.000 | 0.000 | 0.000 | 1.0E+00 | 1.0E+00 | 48 | 1 |
| 5vs6 | 2 | 0.211 | 0.114 | 1.846 | 6.5E-02 | 1.7E-01 | 48 | 1 |
| 5vs6 | 3 | 0.059 | 0.119 | 0.498 | 6.2E-01 | 1.0E+00 | 48 | 1 |

**Lactobacillus spp. dominance**

1. *Lactobacillus* spp. Dominated

2. Intermediate dominance

3. Devoid of *Lactobacillus* spp.

**Patient Groups**

1=Controls (Term Delivery)

2= Before PPROM

3= After PPROM before erythromycin

4= After PPORM after 48h erythromycin

5= After PPROM after <1 week erythromycin

6= After PPROM after >1 week erythromycin

**Table S3. Linear regression analysis comparing proportion of *Lactobacillus* spp. dominance in paired samples before and after 48h erythromycin treatment.**

| *Lactobacillus* spp. Dominance | estimate | se | t | p | q | n |
| --- | --- | --- | --- | --- | --- | --- |
| 1 | -0.438 | 0.161 | -2.725 | 6.4E-03 | 1.9E-02 | 32 |
| 2 | 0.313 | 0.154 | 2.030 | 4.2E-02 | 6.4E-02 | 32 |
| 3 | 0.125 | 0.168 | 0.745 | 4.6E-01 | NA | 32 |
| continuous | -0.563 | 0.290 | -1.939 | 5.2E-02 | NA | 32 |

**Table S4. Maternal and Neonatal factors in the presence and absence of Chorioamnionitis +/- Funisitis.**

|  | Normal | Chorioamnionitis +/- Funisitis | *P* value |
| --- | --- | --- | --- |
| Number | 15 | Chorioamnionitis -4  Chorioamnionitis + Funisitis- 34  Chorioamnionitis+/-Funisitis- 38 |  |
| Sex  F  M | 7 (47%)  8 (53%) | 18 (47%)  20 (53%) |  |
| Mode of Del  Vaginal  Instrumental  LSCS | 7 (47%)  1 (6%)  7 (47%) | 18 (47%)  3 (8%)  17 (45%) |  |
| Gestation PPROM  (weeks) | 27^+1^ (25^+3^ – 28^+6^) | 26^+3^ (25^+3^- 27^+2^) | 0.43 |
| Gestation Del | 31^+5^ (29^+4^ – 33^+6^) | (27^+3^ (26^+3^- 28^+2^) | 0.009 |
| Latency- PPROM to Del  (days) | 24.7 (6.2-43.2) | 6.8 (5-8.7) | 0.88 |
| Birthweight  (g) | 1827 (1555-2088) | 935 (727-1226) | 0.001 |
| Apgar (min)  1  5  10 | 6.9 (4.7-9.0)  8.8 (7.8-9.8)  9.3 (8.7-9.9) | 5.4 (4.6-6.2)  7.7 (7.0-8.3)  8.7 (8.0-9.4) | 0.049  0.042  0.47 |
| Umbilical Cord  Arterial pH  Venous pH | 7.28 (7.21-7.31)  7.31 (7.29-7.34) | 7.23 (7.21-7.32)  7.33 (7.28-7.44) | 0.39  0.53 |
| Maternal CRP  (mg/dL) | 2.7 (1.4-4.2) | 33.2 (12.9-57.4) | 0.000016 |
| Maternal WCC  (10^9^/L) | 12.2 (9.0-13.8 | 17.0 (12.7-21.1) | 0.0016 |
| Neonatal CRP  (mg/dL) | 0.3 (0.2-3.92) | 17.7 (7.0-28.3) | 0.0076 |

Only data from cases where placenta and fetal membranes were sent for histology is included. Placental tissue was not sent for histology in 34 cases. Data presented as median (interquartile range) or number (%) . *P*-values calculated by Mann Whitney U unless stated otherwise

**Table S5. Linear regression analysis comparing proportion of *Lactobacillus* spp. dominance in cases with and without choriomanionitis +/- funisitis.**

| Dysbiosis Group | estimate | se | t | p | q | n | paired |
| --- | --- | --- | --- | --- | --- | --- | --- |
| 1 | 0.483 | 0.146 | 3.298 | 9.7E-04 | 2.9E-03 | 52 | 0 |
| 2 | -0.162 | 0.158 | -1.028 | 3.0E-01 | 3.0E-01 | 52 | 0 |
| 3 | -0.321 | 0.170 | -1.884 | 6.0E-02 | 8.9E-02 | 52 | 0 |
| continuous | 0.804 | 0.276 | 2.917 | 3.5E-03 |  | 52 | 0 |

|  | Early Onset Neonatal Sepsis | No Early Onset Neonatal Sepsis | *P* value |
| --- | --- | --- | --- |
| Number | 16 | 56 |  |
| Sex  F  M | 9 (56%)  7 (44%) | 26 (46%)  30 (54%) | 0.46 |
| Mode of Del  Vaginal  Instrumental  LSCS | 7 (44%)  4 (25%)  5 (31%) | 24 (43%)  6 (11%)  26 (46%) | 0.24 |
| Gestation PPROM  (weeks) | 27^+1^ (25^+3^ – 28^+6^) | 29^+3^ (28^+1^- 30^+4^) | 0.07 |
| Gestation Del  (weeks) | 27^+6^ (26^+1^ – 29^+3^) | 31^+2^ (30^+1^- 32^+2^) | 0.0046 |
| Latency (days)  (PPROM-Del) | 5 (2.7-7.4) | 10.5 (2.3-18.7) | 0.59 |
| Birthweight  (g) | 1100 (790-1411) | 1707 (1519-1896) | 0.0037 |
| Apgar (min)  1  5  10 | 5.7 (4.5-6.8)  7.8 (7.3-8.3)  8.8 (8.1-9.4) | 6.8 (6.1-7.5)  8.5 (8.0-9.0)  9.2 (8.8-9.7) | 0.046  0.01  0.03 |
| Umbilical Cord  Arterial pH  Venous pH | 7.26 (7.21-7.30)  7.33 (7.30-7.37) | 7.25 (7.22-7.28)  7.30 (7.28-7.33) | 0.93  0.29 |
| Placental Histology  Normal  Chorioamnionitis +/- Funisitis  Not sent | 0 (0%)  14 (88%)  2 (12%) | 14 (25%)  21 (37.5%)  21 (37.5%) | 0.0045* |
| Maternal CRP  (mg/dL) | 42.84 (26.8-58.8) | 20.6 (13-28) | 0.0008 |
| Neonatal CRP  (mg/dL) | 40.87 (21.6-58.4) | 1.72 (0.79-2.6) | 1.79 x 10^-8^ |
| Neonatal Duration of Antibiotics (h) | 120 (112-128) | 49 (41-57) | 3.12 x 10^-8^ |

**Table S6. Maternal and Neonatal Factors associated with Early Onset Neonatal Sepsis**

Cases of intrauterine death and cases with insufficient neonatal metadata were removed from the analysis (n=15). Data presented as median (interquartile range) or number (%). *P* values represent comparison between cases of EONS and No EONS, categorical data analysed by Fisher’s Exact and continuous non-parametric data by Mann Whitney unless stated otherwise. *Significance calculated comparing cases where placental histology was sent.
